# Peer-assisted learning after onsite, low-dose, high-frequency training and practice on simulators to prevent and treat postpartum hemorrhage and neonatal asphyxia: A pragmatic trial in 12 districts in Uganda

**DOI:** 10.1371/journal.pone.0207909

**Published:** 2018-12-17

**Authors:** Cherrie Lynn Evans, Eva Bazant, Innocent Atukunda, Emma Williams, Susan Niermeyer, Cyndi Hiner, Ryan Zahn, Rose Namugerwa, Anthony Mbonye, Diwakar Mohan

**Affiliations:** 1 Technical Leadership Office, Jhpiego, Baltimore, Maryland, United States of America; 2 Jhpiego, Kampala, Uganda; 3 University of Colorado School of Medicine, Aurora, Colorado, United States of America; 4 Emory University Rollins School of Public Health, Atlanta, Georgia, United States of America; 5 Save the Children International, Kampala, Uganda; 6 Makerere School of Public Health, Former Director General of Health Services Ministry of Health Kampala, Uganda; 7 Johns Hopkins Bloomberg School of Public Health, Baltimore, Maryland, United States of America; Uppsala University, SWEDEN

## Abstract

An urgent need exists to improve and maintain intrapartum skills of providers in sub-Saharan Africa. Peer-assisted learning may address this need, but few rigorous evaluations have been conducted in real-world settings. A pragmatic, cluster-randomized trial in 12 Ugandan districts provided facility-based, team training for prevention and management of postpartum hemorrhage and birth asphyxia at 125 facilities. Three approaches to facilitating simulation-based, peer assisted learning were compared. The primary outcome was the proportion of births with uterotonic given within one minute of birth. Outcomes were evaluated using observation of birth and supplemented by skills assessments and service delivery data. Individual and composite variables were compared across groups, using generalized linear models. Overall, 107, 195, and 199 providers were observed at three time points during 1,716 births across 44 facilities. Uterotonic coverage within one minute increased from: full group: 8% (CI 4%‒12%) to 50% (CI 42%‒59%); partial group: 19% (CI 9%‒30%) to 42% (CI 31%‒53%); and control group: 11% (5%‒7%) to 51% (40%‒61%). Observed care of mother and newborn improved in all groups. Simulated skills maintenance for postpartum hemorrhage prophylaxis remained high across groups 7 to 8 months after the intervention. Simulated skills for newborn bag-and-mask ventilation remained high only in the full group. For all groups combined, incidence of postpartum hemorrhage and retained placenta declined 17% and 47%, respectively, from during the intervention period compared to the 6‒9 month period after the intervention. Fresh stillbirths and newborn deaths before discharge decreased by 34% and 62%, respectively, from baseline to after completion, and remained reduced 6‒9 months post-implementation. Significant improvements in uterotonic coverage remained across groups 6 months after the intervention. Findings suggest that while short, simulation-based training at the facility improves care and is feasible, more complex clinical skills used infrequently such as newborn resuscitation may require more practice to maintain skills.

**Trial Registration**: ClinicalTrials.gov NCT03254628.

## Introduction

Postpartum hemorrhage (PPH) remains the leading cause of maternal death globally, causing nearly 45,000 deaths per year [1]. Despite evidence that active management of the third stage of labor (AMTSL) reduces the incidence of PPH, AMTSL is not widely practiced according to guidelines [2]. Intrapartum complications account for 23% of all neonatal mortality [3]. Among neonates, an estimated 3‒6% need positive pressure ventilation at birth [4]. An urgent need exists to improve emergency obstetric and neonatal resuscitation skills, particularly in sub-Saharan Africa, where a skilled provider is present at approximately 60% of births, and less than 10% of newborns are attended by providers with resuscitation skills [4, 5].

Traditional in-service education, such as for emergency obstetric and newborn care (EmONC), removes providers from their facilities for 1 to 3 weeks and does not always improve provider performance [6, 7]. In low-volume facilities where providers have fewer opportunities to resuscitate newborns, repeated refresher trainings are needed [8, 9]. Short, simulation-based team learning at the job site—sometimes called “low-dose, high-frequency” (LDHF) training—followed by deliberate practice, improves learning outcomes and provider performance more than “training only” interventions [10–12]. Skills practice after training is best facilitated by designated peers. Peer-assisted learning (PAL) is a cooperative teaching and learning strategy in which learners are active, equal partners. It can be used to facilitate skills practice. While rarely used in sub-Saharan Africa and other low-resource settings, PAL has been a common feature of medical education in the West [13].

The Helping Mothers Survive (HMS) and Helping Babies Survive (HBS) training programs use targeted, simulation-based team learning to promote maximal improvement in and retention of clinical knowledge, skills, and attitudes. The Helping Babies Breathe (HBB) module in the HBS suite has been studied for provider performance and health outcomes [14–16]. HMS Bleeding after Birth (BAB) has been evaluated for knowledge and skills transfer but needs to be evaluated for clinical performance and health outcomes [17–19]. Often, care for the woman and newborn is provided by a single provider and therefore it is essential that training for both be linked. This paper describes a novel intervention that paired facility-based LDHF training in maternal and newborn care and added deliberate practice supported by PAL. Previous evaluations of LDHF training have occurred in controlled conditions in low-resource settings [8, 14, 16, 19]. Under typical research conditions, training with ongoing practice can be implemented with great fidelity; however, fidelity to training and ongoing practice is unknown in real-world conditions. More studies are needed that address elements of implementation research [20]. Hence, the effectiveness of a PAL intervention after initial LDHF training needs to be tested under routine implementation if it is to be sustainable.

This paper describes a trial in near real-world conditions assessing facility-based LDHF training using BAB and HBB with PAL after training to support skills practice at facilities offering childbirth services in Uganda. The objective of the study was to assess the effectiveness of three different strategies for delivering LDHF plus PAL in sustaining improvements in provider skills and clinical performance for prevention and management of PPH and management of birth asphyxia. We demonstrated that LDHF with PAL was effective in improving and maintaining these skills and competencies.

## Materials and methods

### Study design and selection of districts

The study design was a pragmatic, cluster-randomized trial conducted from January 2014 to October 2015 in Western and Eastern Uganda. The cluster-based design was used because the intervention was designed to be delivered at the district level to facilitate referral of complicated cases from lower-level facilities to higher-level facilities. In addition, at the district level, supportive supervision of clinical staff is already offered by the Ministry of Health, therefore, all providers on the labor ward needed to be included in the intervention. All public health facilities with maternities in 12 districts participated. The selection of locations was as follows: we selected two regions ranked in the middle of government rankings for skilled birth attendance and under-5 mortality. Districts were excluded from consideration if they had known ongoing or recent maternal or newborn health initiatives or a facility average of less than one birth per day. This resulted in six districts in each region; all were retained, for a total of 12 districts. Meetings were held with District Health Management Teams to invite districts to participate in the study and request their approval to train and observe providers and collect facility data. All districts agreed to participate prior to randomization of districts to study groups. The primary outcome of the study was the proportion of women receiving a uterotonic within 1 minute of vaginal birth. At the start of the study, we did not consider this study of training methodology to fit the definition of a clinical trial so we did not register until the study concluded. The study is now registered on clinicaltrials.gov #NCT03254628.

### Study setting

The Ugandan public sector health system offers maternity services in hospitals and health center (HC) II–IV. Deliveries typically occur in higher volumes at HC III and IV, with HC IV usually providing cesarean delivery; in some districts, HC IIs conduct deliveries. One regional referral hospital was included in the full intervention group.

### Randomization

**Fig 1:**
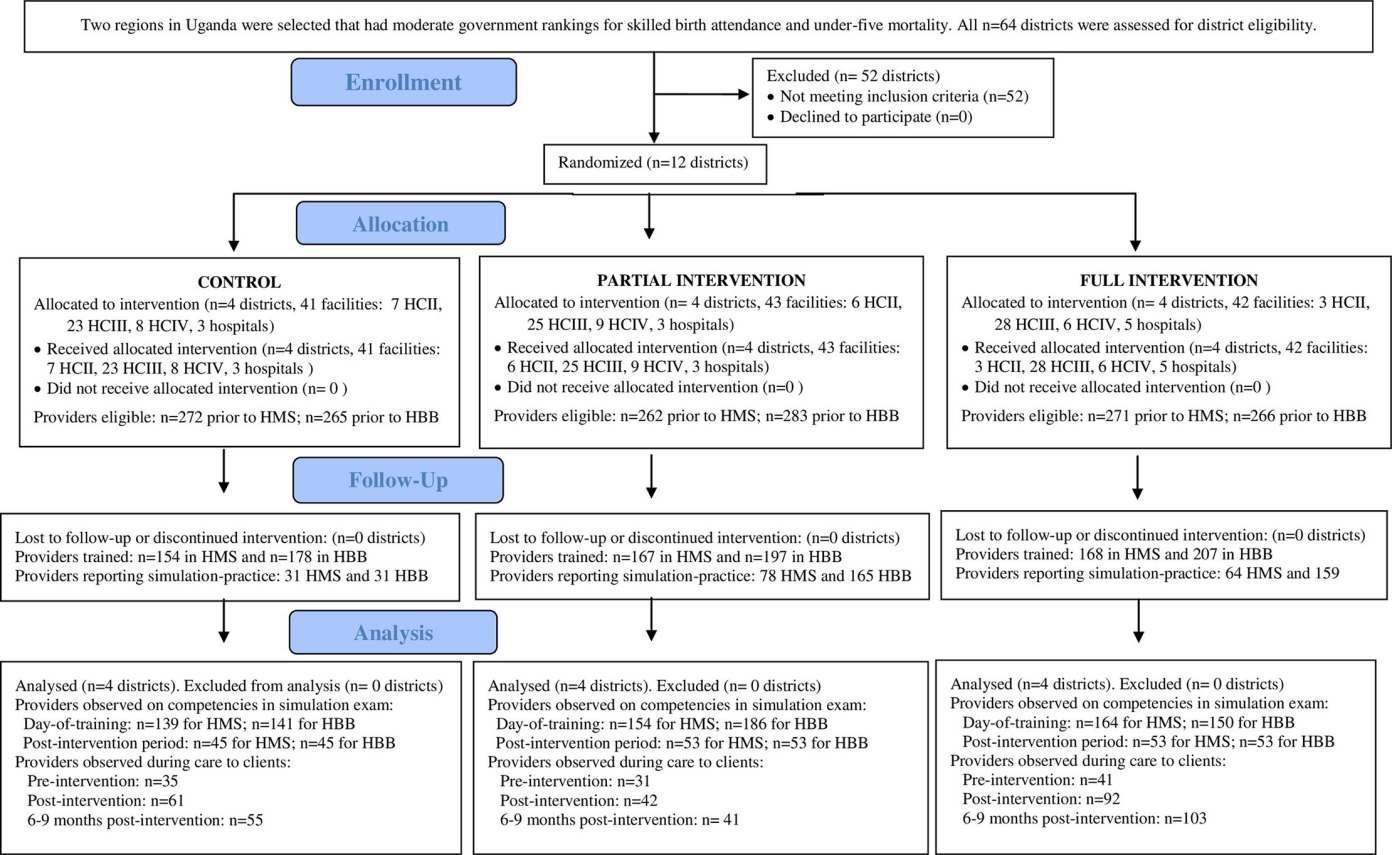
Flow diagram: Peer-assisted learning to sustain provider performance after onsite low-dose training.

In each region, six districts were divided into two groups of three, matched as much as possible on facility-based birth volume and composition of facilities. Within each triplet, districts were randomly assigned by study staff to one of three study groups. Where there was no district hospital, private not-for-profit facilities that functioned as the district hospital were also included, resulting in a total of 126 facilities. One facility dropped out after 6 months because it discontinued intrapartum services (Fig 1).

### Intervention

We provided simulation-based training in management of PPH and neonatal resuscitation to all facilities in all study groups using the BAB training module in June 2014 and the HBB training module in September 2014. Training was delivered as two separate one-day team trainings at all facilities with all providers on the labor ward invited to participate. No providers declined. To implement this training, two clinically active midwives with training experience were selected from each district to be trainers. All 24 trainers passed ModCAL for Training Skills, a computer-based course that helps learners become more effective trainers. Trainers were subsequently trained in BAB and HBB and were mentored by a master trainer during their first facility-based training of each module prior to implementation. BAB and HBB trainings were purposefully spaced 3 months apart to allow for consolidation of learning by both trainers and providers.

At the end of each training, all providers were instructed to practice specific scenarios using simulators for 10–15 minutes every week for 8 weeks, and then combined maternal and newborn scenarios for 4 weeks for a total of 20 weeks of practice. Simulators were left at each facility for skills practice.

**Fig 2:**
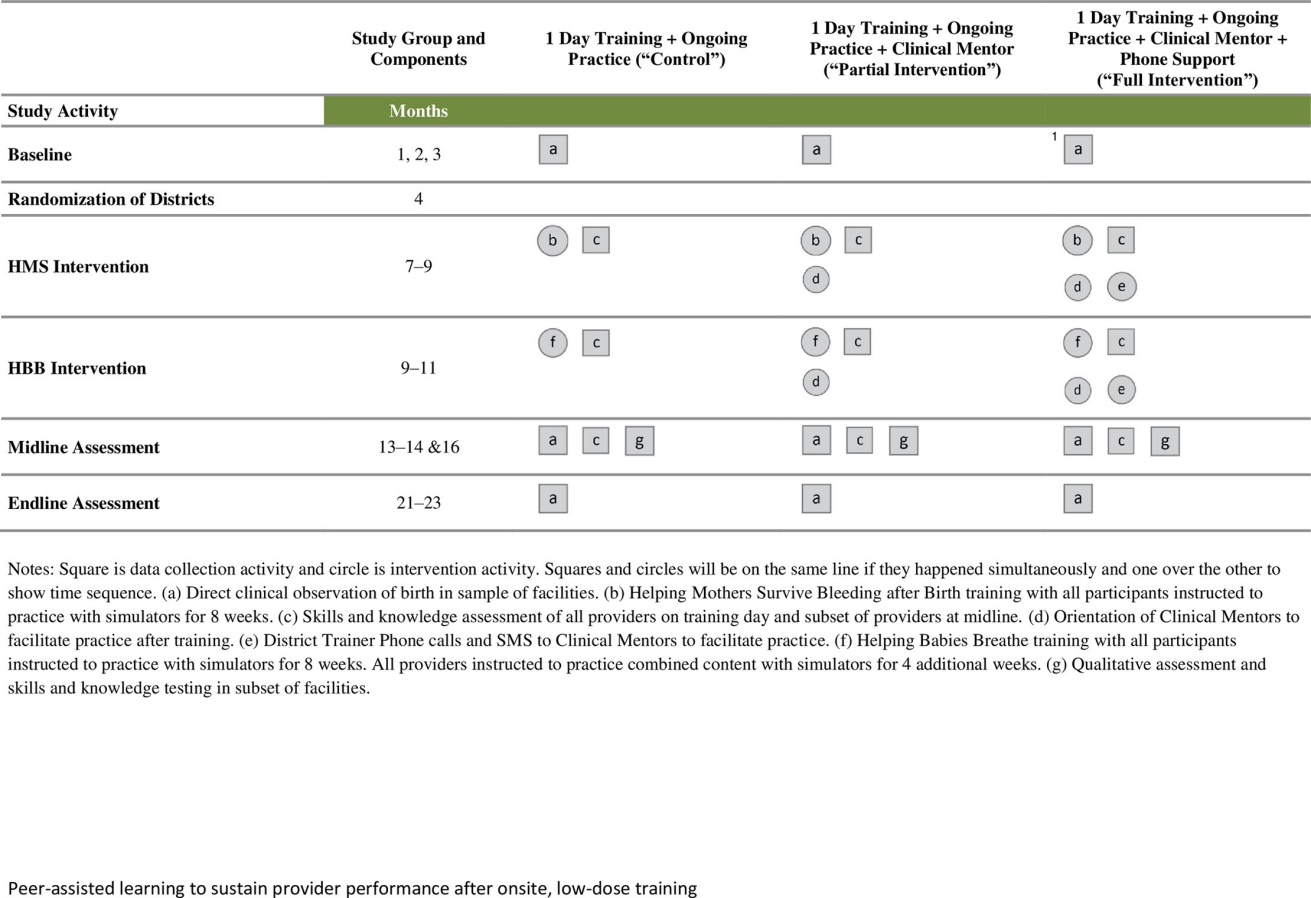
Diagram of intervention elements and assessments, by time point and study group, Uganda BAB and HBB Study.

We compared the training intervention alone (control group) with the same intervention with an added PAL component as follows. The full intervention and partial intervention groups received the training and practice intervention as described above. In addition, district trainers selected Clinical Mentors from each facility and oriented them to a PAL role to support onsite practice after training. Clinical Mentors were practicing midwives who were tasked with organizing and leading brief, structured practice sessions onsite, once per week with fellow providers in addition to performing their clinical duties. In the full intervention group, as an additional element, district trainers made telephone calls to remind Clinical Mentors to facilitate practice (Fig 2).

### Assessment

#### Objective structured clinical examination

Data collection for objective structured clinical examination (OSCE) was done by district trainers from outside the study districts, who received training on the OSCE assessment. The OSCE instruments used were validated BAB and HBB learner assessments [16, 17] to test skills of all providers before and after training using simulators. The BAB OSCE tested provider skills for AMTSL, and the HBB OSCE assessed newborn resuscitation.

Three to 6 months after the intervention period ended (April 2015), we tested a sample of providers in BAB and HBB knowledge and OSCEs to determine whether competencies were retained over time (Fig 2). We conducted OSCEs in six districts in Western and Eastern regions with representation from each study group. All district hospitals and five or six health centers per district were selected, for a total of seven facilities per district. The 42 facilities selected had higher numbers of providers to increase the odds of conducting OSCEs on a trained provider. We found fewer trained providers onsite than expected; thus, 5 facilities were added to reach the desired number of providers. The sample size for post-intervention OSCEs was based on an expected drop in the pass rate in the control group at midline and maintenance at immediate post-training levels for providers in the intervention groups. At midline, the OSCE pass rate was expected to be 45% in the control group and 80% in each intervention group. We set power at 0.80, one-sided significance level of 0.025, and intra-class correlation of 0.1. The sample size calculation called for 162 providers or 54 providers in each study group.

We examined the proportion of providers passing the OSCEs. For BAB, providers had to perform nine of 12 items correctly. For HBB, providers had to perform four mandatory items correctly and at least 14 of 18 items.

#### Direct clinical observation

Direct clinical observation (DCO) occurred at three time points: baseline (before intervention), midline (6 months after the start of the intervention, when activities were complete), and endline (6 months later). DCO data came from 42 facilities that had more than one birth per day, with seven facilities selected in each of six districts and one district per study group in each region.

The primary outcome was the proportion of women receiving a uterotonic in correct dose within 1 minute of vaginal birth. Correct uterotonic use was chosen because the primary author had designed the training package for BAB with an understanding that AMTSL was not widely practiced to standard [2]. Therefore, one of the main goals of this study was to evaluate BAB to understand if the training improved provider performance for this vital component of AMTSL. This indicator was used to determine the sample size of observations. Secondary outcomes included two composites. Care of the mother (coded “1” if 6+ of 7 items were done) included checking for a second baby before administering the uterotonic, giving the correct dose of uterotonic within 1 minute, giving the uterotonic before cord clamping (per time-stamp), checking uterine tone upon delivery of the placenta, visually assessing completeness of the placenta and membranes within 15 minutes of delivery of the placenta, assessing for lacerations, and visually assessing bleeding within 1 minute of delivery of the placenta. Care of the newborn (coded “1” if all 4 items were done) included immediately covering the newborn, including the head, with dry blanket; visually assessing the newborn breathing a second time; placing the baby skin to skin or wrapping the baby in dry towel; and encouraging the mother to breastfeed within 1 hour.

A sample size of 189 births was calculated for each group for midline and endline based on a comparison of proportions to detect a 15% difference with 80% power, type I error of 0.05, and an intra-class correlation at the provider level of 0.01 for the primary outcome.

Sample size was also estimated for non-inferiority of outcome for administration of uterotonic at endline compared to midline. We hypothesized that at midline the outcome would be 70% in the full group, therefore 378 births were needed in the full intervention group for a midline-to-endline (within-group) non-inferiority calculation with 80% power to detect a non-inferiority margin of -0.10 at a 97.5% one-sided significance level, assuming intra-class correlation of 0.005 based on prior studies [21]. Due to lower prevalence of the outcome at baseline, the sample size was revised prior to the midline data collection to be 408 at midline and 459 at endline.

Midwives from outside facilities who received a one-week training did the observations. The data collection instrument was stored on electronic tablets and the devices’ internal clocks were used to accurately record events to the second, including timing of birth, administration of a uterotonic and delivery of the placenta. Data collectors listed all providers working in each facility’s maternity, and this data was used to estimate training and practice coverage and to account for repeated observations of providers.

#### Practice logs

The mean number of practice sessions per provider was calculated from providers’ recording of their practice sessions on logs. This is reported elsewhere [22].

#### Health outcome data from facility registers

To assess the effect of the intervention on services delivered to clients and health outcomes, we collected routine monthly service delivery statistics from the standard government maternity register and introduced a supplemental register to capture other key indicators. Baseline data from January to March 2014 were collected in May 2014 to serve as baseline. No data were collected for April to June 2014 immediately prior to implementation due to funding constraints. Register data were collected monthly from June 2014, when the intervention began, to September 2015.

Adverse perinatal events were the number of fresh (intrapartum) still births plus neonatal deaths prior to discharge per 1,000 births. The maternal outcomes of interest were proportion of vaginal births with PPH and retained placenta. PPH was defined as estimated blood loss greater than 500mL after delivery of the infant. For the purpose of this study, retained placenta was defined as placental delivery greater than 30 minutes after birth. This definition was used because the intervention was designed to reinforce timely prophylactic uterotonic for prevention of PPH (with in minute) and to train providers to treat retained placenta of more than 30 minutes with repeating oxytocin and controlled cord traction. We wanted to be able to assess if 1) prophylactic uterotonic decreased retained placenta over 30 minutes and, 2) if training resulted in a repeat dose of oxytocin at 30 minutes. Monthly summary forms were obtained directly from health facility records officers.

### Data analysis

All analyses were analyzed according to the intention-to-treat (ITT) protocol. For all OSCEs, the individual provider was the unit of analysis. For DCOs, deliveries were the unit of analysis for the maternal indicators and live births were the unit of analysis for the neonatal indicators. The service statistics were reported as a summary aggregate of births or other indicators. Analyses were done using Stata version 15.0 [23].

To estimate the ‘difference in difference’ (differences across time between groups) effect of the intervention for both OSCEs and DCOs, we fit a population-averaged generalized linear model estimated using generalized estimating equations (GEE) with a binomial distribution, logit link and exchangeable correlation structure with Huber-White estimator of variance [23]. In prior exploratory analyses of the DCOs, a random-effect logistic regression model was used to estimate within provider- and within-facility correlations. After running the fully adjusted model, the intra-class correlation (ICC) at facility level was less than 0.1. Given the relatively low correlation at facility level and to estimate the average effect, the primary analysis accounted for clustering within the provider only using GEE. The model included the time point (baseline, midline and endline), study group (comparison group, partial intervention or full intervention) and their interaction term, to assess for differences in change over time by study group. The adjusted model controlled for region (Eastern, Western), facility type (hospital or health center IV, III or II), baseline number of deliveries at the facilities (<100, 100 to 250, 250+) and district. Predicted probabilities from the GEE model and their 95% CI are presented along with adjusted odds ratios.

For the non-inferiority analysis of the primary outcome of correct uterotonic use, we used a generalized linear model with binomial distribution and identity link to estimate risk differences in the full intervention group comparing midline to endline, accounting for clustering in the provider. The 95% confidence intervals for the difference in the proportion of the outcome between midline and endline were interpreted with respect to the non-inferiority margin according to the strategy of Piaggio et al [24]. In examining the difference between midline and endline on the positive outcome, if the upper limit was less than 0.1, then there was non-inferiority of endline compared to midline.

From the data on health outcomes in the monthly facility register, results were presented as proportions, with 95% CIs. Sample size was not powered to detect differences across study groups and outcomes are compared across time points for the baseline (January‒March 2014), program implementation (July‒December 2014) and post-implementation (January‒September 2015) periods.

### Ethical review

This study received ethical approval from the Institutional Review Board of Johns Hopkins Bloomberg School of Public Health (#5383 S2 File), the Research and Ethics Committee of the Makerere University College of Health Sciences School of Public Health (#030), and the Uganda National Council of Science and Technology. All providers consented to participate in the study before training and before observation; Information about the study was read aloud to all providers. Then individually and in private, a provider was asked if there were any questions, and if they consented to the study. Names of consented providers were entered to a listing of participants. Women in labor or their next of kin received the consent information orally and were consented orally prior to observation with this noted in the tablet computer used for data collection.

## Results

### Baseline characteristics

In the baseline period (January to March 2014) the partial group had fewer births (4,379) than the full and control groups (5,590 and 5,456, respectively) (Table 1), lower rates of cesarean births, and higher rates of newborn death than the other two groups. The full group had higher rates of birth asphyxia, defined as Apgar score < 7 (44.3 per 1,000 live births), than did the partial group (15.9) and the control group (24.7), and higher rates of fresh stillbirth (92 per 1,000 live births) than the partial and control groups (38 and 33, respectively).

**Table 1:** Characteristics of study groups at baseline, Uganda.

	Control	Partial	Full
**Prior to Study Start–HMIS**			
Population, 2012 (a)	799,566	632,319	728,327
Facility Deliveries (7/2012‒6/2013) (b)	22,914	19,819	23,028
**Baseline data collected by study team (Jan. to March 2014)**			
*Maternal outcomes at study facilities*			
Deliveries	5,456	4,379	5,590
Live births	5,186	4,389	5,322
Vaginal births	4,857	4,307	4,863
Cesarean sections	568	140	712
Percentage of births as cesarean delivery (c)	10.4	3.2	12.7
Maternal deaths [rate per 1000 deliveries]	6 [1.1]	3 [0.7]	8 [1.4]
*Newborn outcomes at the study facilities*			
Birth asphyxia # [rate per 1000 live births]	128 [24.7]	70 [15.9]	236 [44.3]
Fresh stillbirths # [rate per 1000 deliveries]	33 [6.0]	38 [8.7]	92 [16.5]
Early neonatal deaths # [rate per 1000 live births]	58 [11.2]	71[16.2]	53 [10.0]

HMIS, Health Management Information System

(a) http://en.wikipedia.org/wiki/Districts_of_Uganda. "Population figures from the 2002 census. Sources: 1) Uganda Bureau of Statistics. Statistical Guidance 2013. Kampala, Uganda., Accessed January 19, 2016 at: http://www.ubos.org/onlinefiles/uploads/ubos/pdf%20documents/abstracts/Statistical%20Abstract%202013.pdf

(b) Ministry of Health-Republic of Uganda, Health Management Information system 2. Accessed on October 1, 2013 from http://hmis2.health.go.ug/hmis2/dhis-web-reporting/showDataSetReportForm.action

(c) Percent of births as cesarean delivery is the mean across districts, reported by the Ministry of Health, Republic of Uganda, Annual Health Sector Performance Report 2011/12. Accessed October 1, 2013 from http://health.go.ug/docs/AHSPR_11_12.pdf

### Training of providers

At baseline, there were 271, 262, and 272 eligible providers in the full, partial, and control groups, respectively (Fig 1). We trained 168, 167, and 154 in BAB, in the full, partial, and control groups, respectively, representing 57% to 64% trained per group (Fig 1). Prior to HBB training, there were 266, 283, and 265 eligible providers in the full, partial, and control groups. We trained 207, 197, and 178 providers in HBB in the full, partial, and control groups, representing 67% to 78% per group.

#### Performance on objective structured clinical examinations

**Fig 3:**
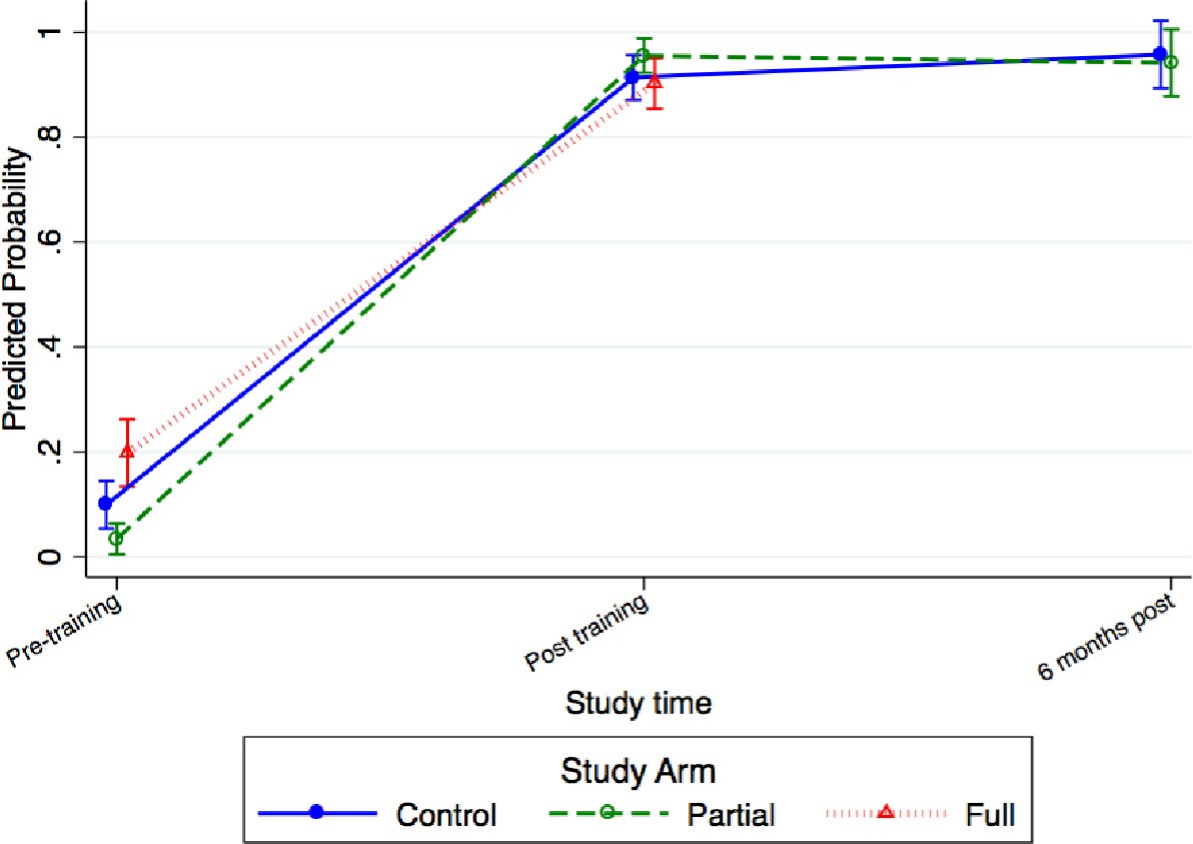
Provider competency by objective structured clinical exam for active management of third stage of labor by group.

Similar trends in the proportion of providers passing the BAB OSCE were observed across groups. The proportion of providers performing to standard for AMTSL increased in the full group from 19.4% (9.5%‒29.3%) pre-training to 90.6% (85.5%‒95.7%) post-training and remained at 95.3% (89.8%‒100%) 7 to 8 months after BAB training and practice concluded (Fig 3). The partial group showed an increase from 3.4% (0.8%‒6%) pre-training to 95.5% (92.1%‒98.9%) post-training and remained at 94.2% (86.4%‒100%) after 7‒8 months, while in the control group the numbers were 9.9% (2.9%‒16.9%), 91.5% (85.3%‒97.7%), and 100%, respectively.

**Fig 4:**
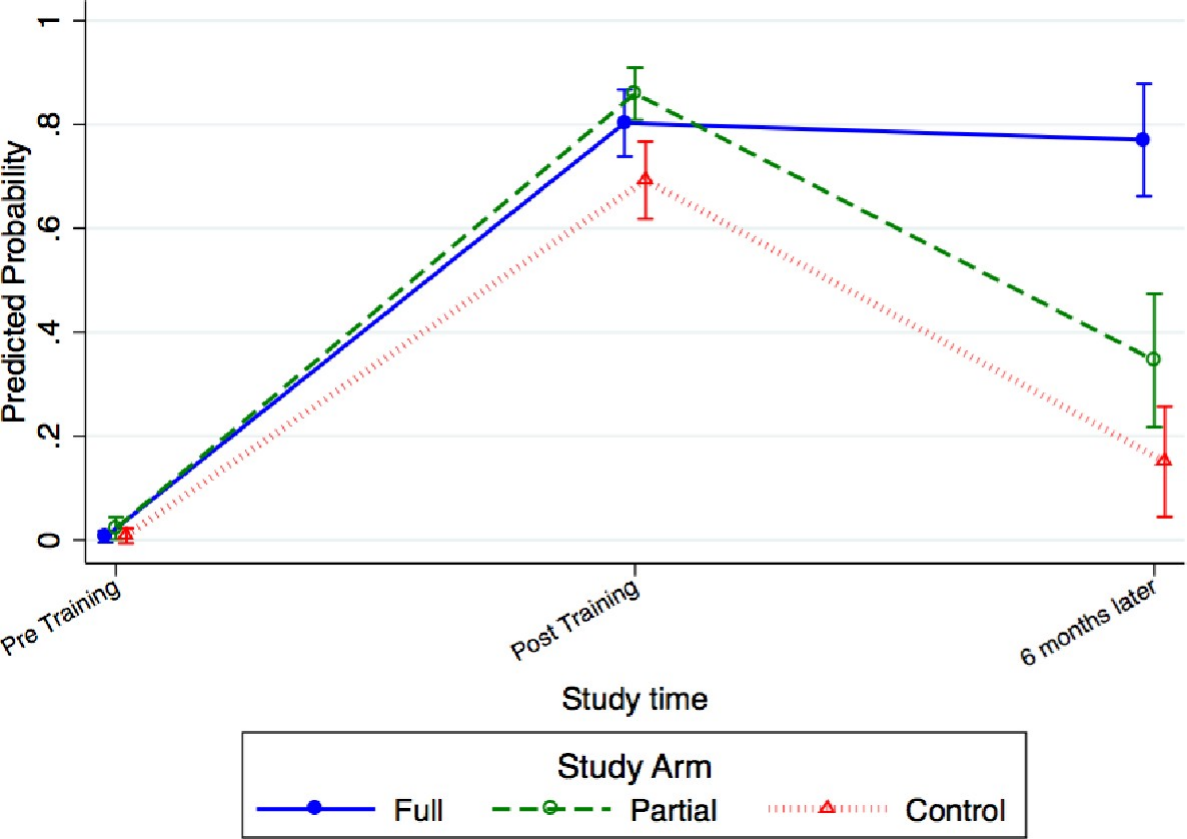
Provider competency by objective structured clinical exam for newborn resuscitation by group.

The proportion of providers performing to standard in the HBB simulated assessments in the full group increased from 0.6% (0‒1.8%) before training to 80% (74%‒86%) after training and remained at 79.2% (66.3%‒92%) 3 to 4 months after HBB training and practice concluded (Fig 4). Performance in the partial group increased from 2.2% (0‒4.7%) pre-training to 86% (80.2%‒91.8%) post-training, but dropped to 35.8% (23%‒48.6%) after 3 to 4 months, while in the control group, it increased from 0.7% (0‒2.1%) before training to 69.5% (56.2%‒82.8%) after training and declining to 15.6% (5.3%‒25.9%) after 3 to 4 months.

#### Performance during direct clinical observation

In the full intervention group, the 938 births observed were 169, 317, and 452 at baseline, midline and endline, respectively. In the full group, 41, 92 and 103 providers were observed providing care in 37, 47, and 44 facilities at baseline, midline, and endline, respectively. The mean number of observations per provider was 3.05 (1‒10) at baseline, 3.19 (1‒12) at midline, and 3.92 (1‒21) at endline.

**Fig 5:**
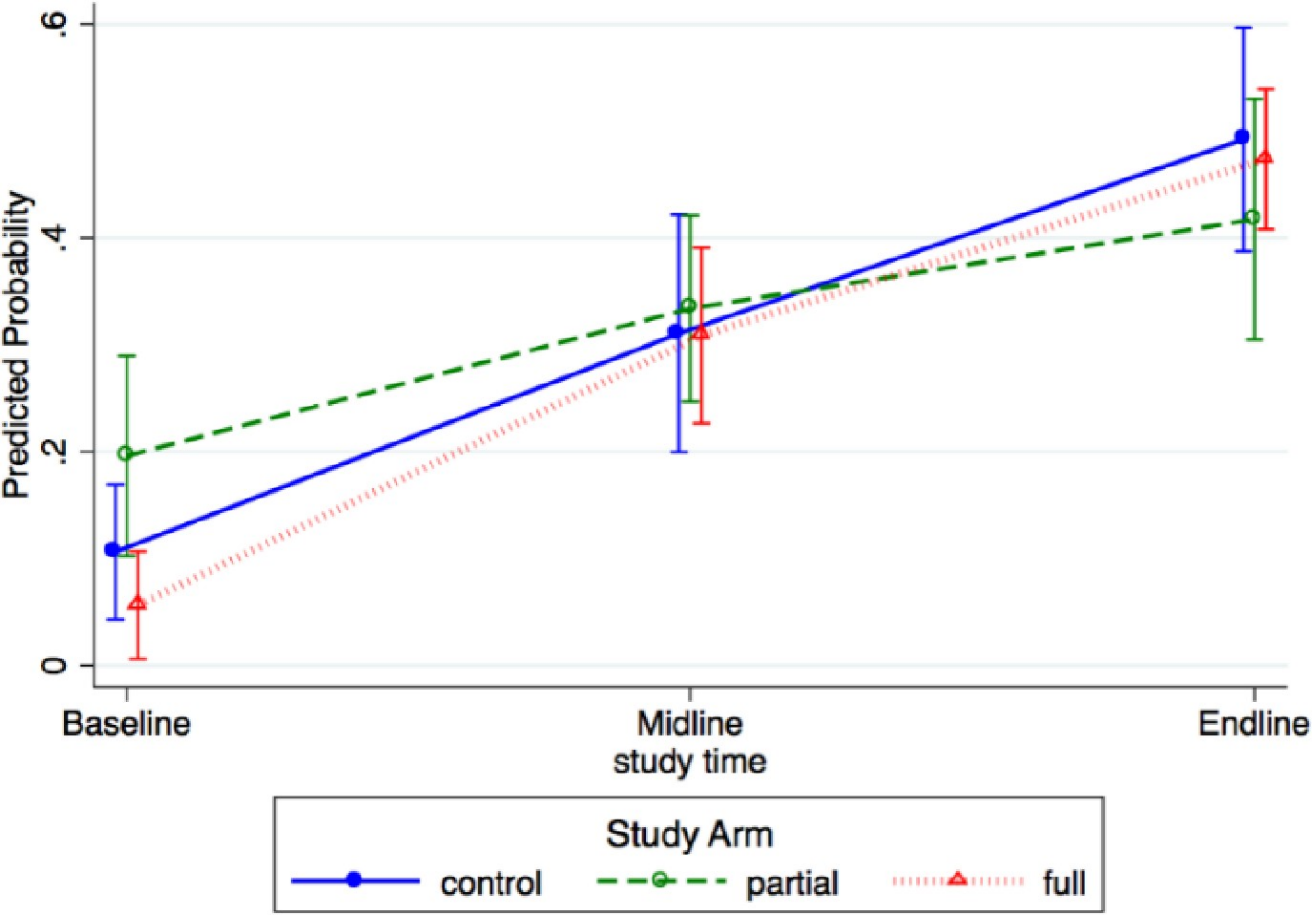
Predicted probability and 95% confidence interval of correct use of uterotonic within 1 minute. (n = 1546 observations of care).

The likelihood of births with prophylactic uterotonic in third stage given correctly within 1 minute in the full group was 6% (CI 1%‒10%) at baseline, 31% (CI 23%‒39%) at midline, and 47% (CI 41%‒54%) at endline (Fig 5 and S1 Table). The difference in the proportion comparing midline and baseline was 25% (22%‒28%) and endline and baseline, 41% (40%‒44%). The adjusted estimated difference in proportions for uterotonic given in correct dose within one minute of birth between midline vs. endline was -.16 (95% CI -.28, -.05), showing that endline results were non-inferior to those of midline.

Comparing changes across groups, the predicted probability of correct uterotonic use in third stage increased in all groups at midline and endline (with overlapping confidence intervals), suggesting no difference across groups.

**Fig 6:**
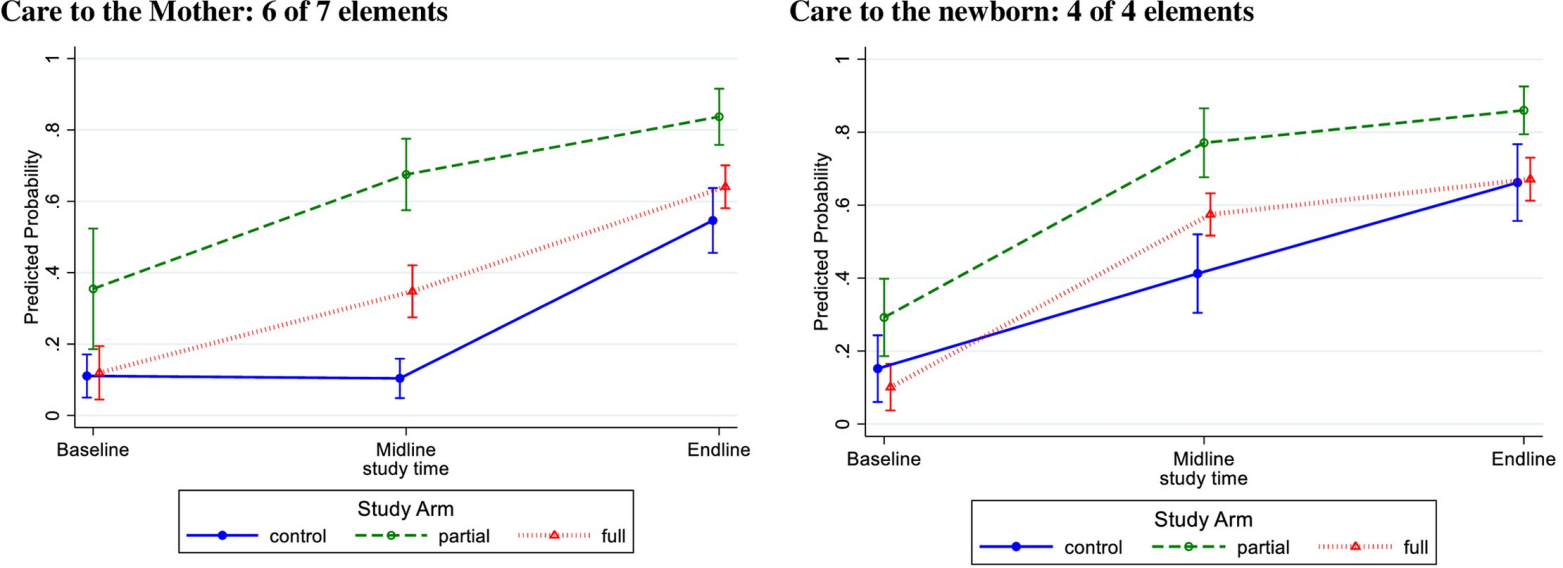
Predicted probability and 95% confidence interval of care to mother and newborn, by study group (n = 1546 observations of care).

The predicted probability of the care of the mother composite outcome was low in control and full groups at baseline and was significantly higher in the partial group than other groups (with wide confidence interval, 18% to 53%) (Fig 6 and S1 Table). All study groups improved. At endline, the partial group had high performance (84%; CI 76%‒92%) and significantly out-performed the control (55%; CI 46%‒64%) and full (64%; CI 58%‒71%) groups.

Similarly, the composite outcome of care of the newborn was low in control and full groups at baseline and was significantly higher in the partial group than other groups (Fig 6 and S2 Table). All groups improved. At endline, the partial group had high performance (88%; CI 80%‒93%) and significantly out-performed the control (66%; CI 56%‒77%) and full (67%; CI 61%‒73%) Comparing providers’ performance at endline compared to baseline, all groups show improvements. The full intervention group had large gains in odds of providers’ performing the care activities in the composite outcomes but these changes were not statistically different from the other two groups (Table 2).

**Table 2:** Effects of Intervention on service delivery outcomes comparing baseline to midline and endline according to regression models, (n = 1546 observations of care).

Outcome & Study group	Adjusted Odds Ratios: Midline to Baseline		Adjusted Odds Ratios: Endline to Baseline	
	(95%CI)	p-value	(95%CI)	p-value
**Uterotonic given in correct dose within one minute**				
Comparison	3.8 (1.7, 8.6)	.001	10.1 (4.5, 22.6)	< .001
Partial Intervention	2.2 (1.0, 4.5)	.039	3.1 (1.6, 5.9)	.001
Full Intervention	6.4 (2.5, 16.4)	< .001	14.5 (6.1, 34.5)	< .001
**Care to mother (b)**				
Comparison	0.8 (0.4, 1.7)	0.590	10.0 (4.7, 21.2)	<0.001
Partial Intervention	4.4 (2.1, 9.6)	<0.001	13.3 (4.7, 37.4)	<0.001
Full Intervention	4.1 (1.9, 9.0)	<0.001	15.9 (7.0, 36.5)	<0.001
**Care to newborn (c)**				
Comparison	5.1 (2.1,12.4)	<0.001	16.6 (5.5, 50.7)	<0.001
Partial Intervention	8.8 (3.9,20.0)	<0.001	17.5 (8.2, 37.5)	<0.001
Full Intervention	14.7(6.5,32.9)	<0.001	24.9 (10.8, 57.1)	<0.001

### Health outcomes from facility registers

**Fig 7:**
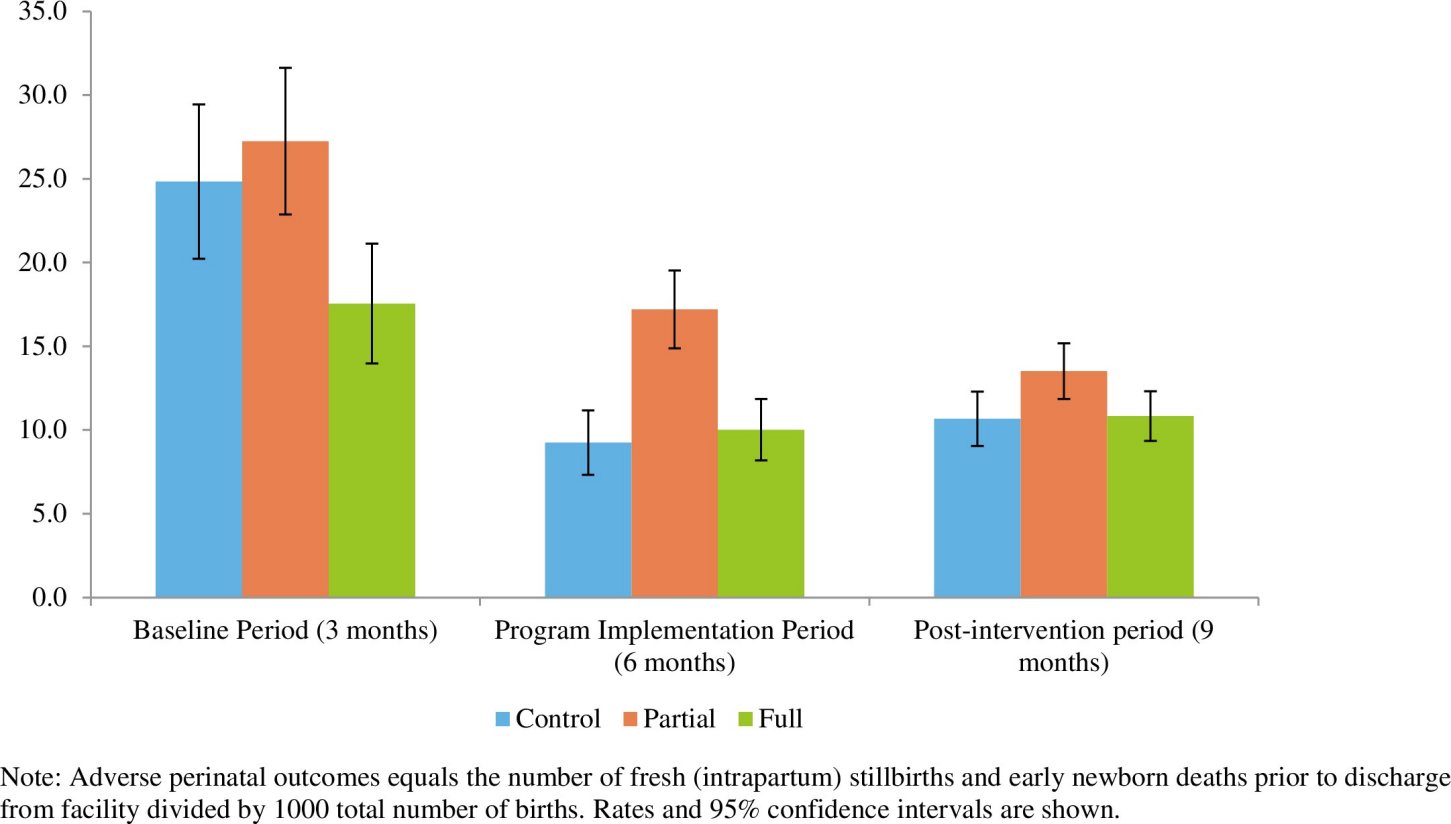
Adverse perinatal outcomes per 1,000 births. (n = 125 facilities).

Across all facilities and all groups combined, the study showed a 17% reduction in incidence of PPH and 47% reduction in retained placenta from during the intervention period compared to subsequent 6–9 month period. Adverse perinatal outcomes decreased significantly from baseline to after the intervention was complete and remained at the reduced levels post-implementation (Fig 7). There was a 34% reduction in fresh stillbirths and a 62% reduction in newborn deaths before discharge for the same period. There were no significant differences across study groups in the reductions in PPH, retained placenta or adverse perinatal events.

## Discussion

This is the first pragmatic trial combining LDHF training interventions for prevention and management of PPH and birth asphyxia using facility-based, hands on learning followed by PAL. This study was designed to determine whether deliberate skills practice through PAL after onsite training improved provider performance. Results show improved and sustained skills during testing for AMTSL and improved delivery of care through clinical observation. Skills for AMTSL during simulation remained high 7–8 months after the BAB intervention was complete, even in the group without PAL to support practice suggesting that this skill that may be ‘practiced’ enough during routine care to maintain competency. However, for neonatal resuscitation including bag-mask ventilation, which is performed infrequently compared to AMTSL, only the full intervention group—which had the greatest support for ongoing practice through reminder phone calls to practice—maintained provider performance during testing 3‒4 months after the HBB intervention was complete. In addition, we demonstrate improvements in patient care, which continued to improve for many indicators, even in the post intervention period. Care improved in all groups on the primary outcome, correct uterotonic delivery within 1 minute of birth (per timestamp). Composite scores of care to the mother and newborn were significantly higher at endline for the intervention groups with PAL compared to the control. All facilities in the intervention groups had a Clinical Mentor to facilitate deliberate practice—the PAL approach—resulting in more skills practice than reported in the control group [22].

This study provides an “adequacy” level of evidence [25] for the effectiveness of onsite, LDHF simulation-based training on reducing incidence of PPH, retained placenta, and adverse perinatal events; however, the study was not powered to detect differences in health outcomes across groups.

Our finding that onsite, simulation-based training in teams can improve learning outcomes and directly observed care for key clinical skills is consistent with those of other studies [11, 15]. However, our intervention is unique with the addition of deliberate skills practice using PAL.

Relating to clinical performance in low-resource settings, some studies have demonstrated an improvement in knowledge and skills after simulation-based training without measuring or reporting improvement in clinical practice or service delivery to clients [14, 19]. Or, studies have shown improved clinical practice at low geographic coverage [15] or no improvement in care at all [6, 26]. For example, Varghese et al implemented facility-based training followed by team drills every 2 months in four facilities in India with no improvement in diagnosis or treatment of maternal and newborn complications [26]. This suggests that the type of intervention as well as the “dose” and whether the intervention is repeated may be critical.

This study had several strengths. To our knowledge, no studies exist of large-scale implementation of facility- and simulation-based training for PPH and newborn resuscitation in developing countries. The system-wide rollout of onsite, LDHF simulation-based training with PAL was implemented in near real-world conditions at public facilities with government trainers and providers, and was designed to be sustainable and scalable. Additional strengths include measurement of outcomes along the impact pathway—skills during simulation, observed care, and health outcomes [10]. We assessed effectiveness in terms of gain and retention over time of important skills, in contrast to other studies that stop evaluation immediately after training [27]. Lastly, the intervention was deployed district-wide with an intent to reach all providers. In a district, health centers already refer cases to hospitals and collaborate, therefore, our study was designed to strengthen the existing system. Our analyses accounted for clustering. In the sample size calculation for OSCE data, we used a ICC of 0.1. In the DCO data, in the sample size calculation of uterotonic given, we used ICC of 0.01 in the superiority analysis and 0.005 in the non-inferiority analysis as suggested by Piaggio et al [24]. In exploratory random-effect logistic regression models, once the provider-level clustering was taken into account, the facility-level clustering was 0.1. Given this low correlation and in order to estimate the population-averaged effect, the main analysis comparing study groups on the change in uterotonic given was done in GEE models accounting for clustering only in the provider.

The study had several limitations. We randomized at the district level as all facilities in the district were to receive the same intervention. Despite matching districts on facility composition and volume of services, study groups differed on baseline characteristics in both measurable and unmeasurable ways. The intervention groups started out with higher adverse event rates than the control. For the full intervention group, this was probably attributable to the inclusion of a regional hospital. The partial group had lower cesarean births perhaps due to differences in the health facilities included in this group which we were unable to explain adequately. Management of neonatal asphyxia and postpartum hemorrhage are relatively rare events; the care performed during those cases was not able to be assessed and compared across study groups with adequate power to detect a difference in provider performance or incidence of adverse maternal and perinatal outcomes. In addition, mortality outcomes are from the health management information system data and may suffer from problems with completeness and representativeness [28]. In this trial, blinding or masking to conceal study groups from participants or study staff was not possible. For observations of care, the Hawthorne effect—providers acting differently when observed than they would normally act—may have existed, but this was minimized due to observers spending multiple days in each facility. The decline in PPH, retained placenta, fresh stillbirth, and early neonatal death could have been related to secular trends; however newly published research has shown similar large declines in fresh stillbirth and early neonatal death rates following a LDHF and PAL intervention [29].

### Global implications

LDHF simulation training of the entire team onsite, coupled with ongoing support for deliberate practice through PAL is feasible and effective in changing provider performance in a low-resource setting. The study suggests that different clinical skills may require varying levels of ongoing practice or retraining for maintenance [8, 30]. National and international policymakers and stakeholders are increasingly calling for performance-enhancing methods to improve maternal and newborn outcomes. In 2016, the World Health Organization published standards for improving quality of maternal and newborn care which recommends annual in-service training for all providers and monthly team drills [31].

In surveyed health systems of seven countries in sub-Saharan Africa to understand the association between in-service training and supervision with the quality of observed antenatal and sick child care [6], Leslie et al found that traditional training and supervision was insufficient to meaningfully improve care. Global maternal health experts have requested new approaches be explored to improve clinical skills after training and that multicenter trials are needed to determine effectiveness of training interventions [7, 32]. Related to hemorrhage specifically, research priorities of international stakeholders to improve maternal health outcomes between 2015 and 2025 included the priority to “evaluate the effectiveness and cost of training interventions for frontline healthcare workers” to detect, manage and refer women with PPH [33]. Simulation-based training combined with practice sessions was superior to traditional training for many clinical skills [11] and multi-professional training interventions for emergency obstetric care in low-resource settings using realistic but simple equipment to simulate obstetric emergencies where providers work, resulted in better coverage at lower cost [34].

The dose and frequency of specific training interventions are important to specify in an intervention, in addition to the method of delivery–didactic vs hands-on simulation and practice, team-based vs individual, facility-based vs workshop [35]. For a training intervention to change provider behavior for any given skill, the “dose” of the intervention is key. Complex but less frequently performed skills (i.e. bag and mask ventilation of the newborn) will require a larger “dose” of training, practice and support. This is compared to AMTSL, which is simpler and should be performed at every birth, suggesting deliberate practice is likely not required. Deliberate practice and simulation after training requires ongoing support that can be assisted through PAL. Specifically regarding dose and frequency of interventions to improve provider performance, the authors suggest more thought is needed as to what the appropriate dose, frequency and modalities are as defined by the targeted clinical skill or competency. Further research is needed to understand the cost effectiveness of this approach and to explore what additional supports are needed for infrequently used but critical skills. This approach for prevention and management of PPH and birth asphyxia can be implemented at the district level, in all facilities conducting birth, in a short time span with an intervention period of 4 to 6 months. This intervention could be scaled up nationally to produce further declines in PPH, retained placenta, fresh stillbirth and early neonatal death.

## Supporting information

S1 Table Providers’ care to the mother by study group, direct clinical observations. (DOCX)

S2 Table Providers’ care to the newborn by study group, direct clinical observations. (DOCX)

S1 File C Evans SLAB CONSORT extension for cluster trials checklist. (DOCX)

S2 File C Evans study protocol JHSPH IRB Uganda. (DOCX)

## References

[1] Kassebaum NJ, Bertozzi-Villa A, Coggeshall MS, Shackelford KA, Steiner C, Heuton KR, et al Global, regional, and national levels and causes of maternal mortality during 1990–2013: a systematic analysis for the Global Burden of Disease Study 2013. Lancet. 2014;384(9947):980–1004. 10.1016/S0140-6736(14)60696-6 24797575 10.1016/S0140-6736(14)60696-6 PMC4255481

[2] Bartlett L, Cantor D, Lynam P, Kaur G, Rawlins B, Ricca J, et al Facility-based active management of the third stage of labour: assessment of quality in six countries in sub-Saharan Africa. Bull World Health Organ. 2015;93(11):759–67. 10.2471/BLT.14.142604 26549903 10.2471/BLT.14.142604 PMC4622150

[3] Lawn JE, Blencowe H, Oza S, You D, Lee AC, Waiswa P, et al Every Newborn: progress, priorities, and potential beyond survival. Lancet. 2014;384(9938):189–205. 10.1016/S0140-6736(14)60496-7 . 24853593 10.1016/S0140-6736(14)60496-7

[4] Wall SN, Lee AC, Niermeyer S, English M, Keenan WJ, Carlo W, et al Neonatal resuscitation in low-resource settings: what, who, and how to overcome challenges to scale up? Int J Gynaecol Obstet. 2009;107 Suppl 1:S47–62, S3-4. 10.1016/j.ijgo.2009.07.013 19815203 10.1016/j.ijgo.2009.07.013 PMC2875104

[5] Enweronu-Laryea C, Dickson KE, Moxon SG, Simen-Kapeu A, Nyange C, Niermeyer S, et al Basic newborn care and neonatal resuscitation: a multi-country analysis of health system bottlenecks and potential solutions. BMC Pregnancy Childbirth. 2015;15 Suppl 2:S4 10.1186/1471-2393-15-S2-S4 26391000 10.1186/1471-2393-15-S2-S4 PMC4577863

[6] Leslie HH, Gage A, Nsona H, Hirschhorn LR, Kruk ME. Training And Supervision Did Not Meaningfully Improve Quality Of Care For Pregnant Women Or Sick Children In Sub-Saharan Africa. Health Aff (Millwood). 2016;35(9):1716–24. 10.1377/hlthaff.2016.0261 . 27605655 10.1377/hlthaff.2016.0261

[7] Gulmezoglu AM, Lawrie TA. Impact of training on emergency resuscitation skills: Impact on Millennium Development Goals (MDGs) 4 and 5. Best Pract Res Clin Obstet Gynaecol. 2015;29(8):1119–25. 10.1016/j.bpobgyn.2015.03.018 . 25937556 10.1016/j.bpobgyn.2015.03.018

[8] Mduma E, Ersdal H, Svensen E, Kidanto H, Auestad B, Perlman J. Frequent brief on-site simulation training and reduction in 24-h neonatal mortality—an educational intervention study. Resuscitation. 2015;93:1–7. 10.1016/j.resuscitation.2015.04.019 . 25957942 10.1016/j.resuscitation.2015.04.019

[9] Mosley C, Dewhurst C, Molloy S, Shaw BN. What is the impact of structured resuscitation training on healthcare practitioners, their clients and the wider service? A BEME systematic review: BEME Guide No. 20. Med Teach. 2012;34(6):e349–85. 10.3109/0142159X.2012.681222 . 22578048 10.3109/0142159X.2012.681222

[10] Bluestone J, Johnson P, Fullerton J, Carr C, Alderman J, BonTempo J. Effective in-service training design and delivery: evidence from an integrative literature review. Hum Resour Health. 2013;11:51 10.1186/1478-4491-11-51 24083659 10.1186/1478-4491-11-51 PMC3850724

[11] McGaghie WC, Issenberg SB, Cohen ER, Barsuk JH, Wayne DB. Medical education featuring mastery learning with deliberate practice can lead to better health for individuals and populations. Acad Med. 2011;86(11):e8–9. 10.1097/ACM.0b013e3182308d37 . 22030671 10.1097/ACM.0b013e3182308d37

[12] Rowe AK, Rowe SY, Vujicic M, Ross-Degnan D, Chalker J, Holloway KA, et al Chapter 3: Review of strategies to improve health care provider performance In: Peters DH, El-Saharty S, Siadat B, Janovsky K, Vujicic M, editors. Improving health service delivery in developing countries: from evidence to action DIRECTIONS IN DEVELOPMENT—Human Development. Washington, DC: The World Bank; 2009 p. 101–26.

[13] Secomb J. A systematic review of peer teaching and learning in clinical education. J Clin Nurs. 2008;17(6):703–16. 10.1111/j.1365-2702.2007.01954.x . 18047577 10.1111/j.1365-2702.2007.01954.x

[14] Ersdal HL, Vossius C, Bayo E, Mduma E, Perlman J, Lippert A, et al A one-day "Helping Babies Breathe" course improves simulated performance but not clinical management of neonates. Resuscitation. 2013;84(10):1422–7. 10.1016/j.resuscitation.2013.04.005 . 23612024 10.1016/j.resuscitation.2013.04.005

[15] Msemo G, Massawe A, Mmbando D, Rusibamayila N, Manji K, Kidanto HL, et al Newborn mortality and fresh stillbirth rates in Tanzania after helping babies breathe training. Pediatrics. 2013;131(2):e353–60. 10.1542/peds.2012-1795 . 23339223 10.1542/peds.2012-1795

[16] Singhal N, Lockyer J, Fidler H, Keenan W, Little G, Bucher S, et al Helping Babies Breathe: global neonatal resuscitation program development and formative educational evaluation. Resuscitation. 2012;83(1):90–6. 10.1016/j.resuscitation.2011.07.010 . 21763669 10.1016/j.resuscitation.2011.07.010

[17] Evans CL, Johnson P, Bazant E, Bhatnagar N, Zgambo J, Khamis AR. Competency-based training "Helping Mothers Survive: Bleeding after Birth" for providers from central and remote facilities in three countries. Int J Gynaecol Obstet. 2014;126(3):286–90. 10.1016/j.ijgo.2014.02.021 . 24834851 10.1016/j.ijgo.2014.02.021

[18] Nathan LM, Patauli D, Nsabimana D, Bernstein PS, Rulisa S, Goffman D. Retention of skills 2 years after completion of a postpartum hemorrhage simulation training program in rural Rwanda. Int J Gynaecol Obstet. 2016;134(3):350–3. 10.1016/j.ijgo.2016.01.021 . 27262941 10.1016/j.ijgo.2016.01.021

[19] Nelissen E, Ersdal H, Mduma E, Evjen-Olsen B, Broerse J, van Roosmalen J, et al Helping Mothers Survive Bleeding After Birth: retention of knowledge, skills, and confidence nine months after obstetric simulation-based training. BMC Pregnancy Childbirth. 2015;15:190 10.1186/s12884-015-0612-2 26303614 10.1186/s12884-015-0612-2 PMC4548347

[20] Peters DH, Tran NT, Adam T. Implementation research in health: a practical guide. Geneva, Switzerland: Alliance for Health Policy and Systems Research, World Health Organization; 2013 Available from: http://www.who.int/alliance-hpsr/resources/implementationresearchguide/en/

[21] Adams G, Gulliford MC, Ukoumunne OC, Eldridge S, Chinn S, Campbell MJ. Patterns of intra-cluster correlation from primary care research to inform study design and analysis. J Clin Epidemiol. 2004;57(8):785–94. 10.1016/j.jclinepi.2003.12.013 . 15485730 10.1016/j.jclinepi.2003.12.013

[22] Williams E, Bazant E, Holcombe S, Atukunda I, Namugerwa R, Britt K, Evans C. 'Let us again, practice so that that skill does not disappear': Mixed methods evaluation of simulator-based practice for midwives in Uganda. Human Resources for Health 2018; in press. 10.1186/s12960-019-0350-z PMC6440002 30925890

[23] Gutierrez R, Drukker DM. Citing references for Stata’s cluster-correlated robust variance estimates? 2018; (Online blogpost). College Station, TX: StataCorp LLC Available from: https://www.stata.com/support/faqs/statistics/references/

[24] Piaggio G, Elbourne DR, Altman DG, Pocock SJ, Evans SJ, Group C. Reporting of noninferiority and equivalence randomized trials: an extension of the CONSORT statement. JAMA. 2006;295(10):1152–60. 10.1001/jama.295.10.1152 . 16522836 10.1001/jama.295.10.1152

[25] Habicht JP, Victora CG, Vaughan JP. Evaluation designs for adequacy, plausibility and probability of public health programme performance and impact. Int J Epidemiol. 1999;28(1):10–8. . 10195658 10.1093/ije/28.1.10

[26] Varghese B, Krishnamurthy J, Correia B, Panigrahi R, Washington M, Ponnuswamy V, et al Limited Effectiveness of a Skills and Drills Intervention to Improve Emergency Obstetric and Newborn Care in Karnataka, India: A Proof-of-Concept Study. Glob Health Sci Pract. 2016;4(4):582–93. 10.9745/GHSP-D-16-00143 27993924 10.9745/GHSP-D-16-00143 PMC5199176

[27] Sutton RM, Niles D, Meaney PA, Aplenc R, French B, Abella BS, et al Low-dose, high-frequency CPR training improves skill retention of in-hospital pediatric providers. Pediatrics. 2011;128(1):e145–51. 10.1542/peds.2010-2105 21646262 10.1542/peds.2010-2105 PMC3387915

[28] Burger EH, van der Merwe L, Volmink J. Errors in the completion of the death notification form. S Afr Med J. 2007;97(11):1077–81. . 18250917

[29] Gomez PP, Nelson AR, Asiedu A, Addo E, Agbodza D, Allen C, et al Accelerating newborn survival in Ghana through a low-dose, high-frequency health worker training approach: a cluster randomized trial. Gomez et al. BMC Pregnancy Childbirth. 2018; 18:72 10.1186/s12884-018-1705-5 PMC5863807 29566659

[30] Kc A, Wrammert J, Clark RB, Ewald U, Vitrakoti R, Chaudhary P, et al Reducing Perinatal Mortality in Nepal Using Helping Babies Breathe. Pediatrics. 2016;137(6). 10.1542/peds.2015-0117 . 27225317 10.1542/peds.2015-0117

[31] Standards for improving quality of maternal and newborn care in health facilities. Geneva, Switzerland: World Health Organization, 2016 978 92 4 151121 6.

[32] Kendall T, Langer A. Critical maternal health knowledge gaps in low- and middle-income countries for the post-2015 era. Reprod Health. 2015;12:55 10.1186/s12978-015-0044-5 26044755 10.1186/s12978-015-0044-5 PMC4475304

[33] Souza JP, Widmer M, Gulmezoglu AM, Lawrie TA, Adejuyigbe EA, Carroli G, et al Maternal and perinatal health research priorities beyond 2015: an international survey and prioritization exercise. Reprod Health. 2014;11:61 10.1186/1742-4755-11-61 25100034 10.1186/1742-4755-11-61 PMC4132282

[34] Bergh AM, Baloyi S, Pattinson RC. What is the impact of multi-professional emergency obstetric and neonatal care training? Best Pract Res Clin Obstet Gynaecol. 2015;29(8):1028–43. 10.1016/j.bpobgyn.2015.03.017 . 25937554 10.1016/j.bpobgyn.2015.03.017

[35] Crofts JF, Winter C, Sowter MC. Practical simulation training for maternity care—where we are and where next. BJOG. 2011;118 Suppl 3:11–6. 10.1111/j.1471-0528.2011.03175.x . 22039887 10.1111/j.1471-0528.2011.03175.x

