# Supplementary material for: Peer-assisted learning after onsite, low-dose, high-frequency training and practice on simulators to prevent and treat postpartum hemorrhage and neonatal asphyxia: A pragmatic trial in 12 districts in Uganda
Source: PLoS One. 2018 Dec 17;13(12):e0207909. doi: 10.1371/journal.pone.0207909 (PMC6296740; doi:10.1371/journal.pone.0207909)
Supplement: S1 Table — (DOCX) [file pone.0207909.s001.docx]

**S1 Table****. Providers’ Care to the Mother by Study Group, Direct Clinical Observations**

|  | **Group** | **Baseline**  **(May 2014)** | | **Midline**  **(Jan-Feb 2015)** | | **Endline**  **(Sept-Oct 2015)** | |
| --- | --- | --- | --- | --- | --- | --- | --- |
| **Element:** |  | **N** | **%** | **N** | **%** | **N** | **%** |
| Checks for second baby before giving uterotonic | Control | 98 | 47 | 139 | 47 | 172 | 65 |
|  | Partial | 92 | 73 | 119 | 87 | 135 | 94 |
|  | Full | 114 | 32 | 344 | 65 | 461 | 76 |
| Gives correct dose of uterotonic within 1 min. | Control | 90 | 11 | 108 | 32 | 156 | 51 |
|  | Partial | 90 | 19 | 113 | 31 | 128 | 42 |
|  | Full | 101 | 8 | 322 | 32 | 452 | 50 |
| …within 5 min. | Control | 88 | 68 | 107 | 92 | 156 | 94 |
|  | Partial | 87 | 59 | 111 | 96 | 128 | 97 |
|  | Full | 100 | 74 | 317 | 91 | 452 | 95 |
| Gives uterotonic before cord was cut (based on timestamps) | Control | 104 | 60 | 144 | 60 | 173 | 82 |
|  | Partial | 97 | 59 | 121 | 92 | 138 | 93 |
|  | Full | 116 | 40 | 354 | 76 | 469 | 79 |
| Checks uterine tone upon delivery of placenta | Control | 101 | 83 | 139 | 82 | 172 | 92 |
|  | Partial | 94 | 91 | 121 | 99 | 136 | 99 |
|  | Full | 114 | 82 | 343 | 85 | 465 | 95 |
| Assesses for completeness of placenta and membranes within 15 min. of birth   \|  \| 1 \| 0 \| 8 \| \| --- \| --- \| --- \| --- \| \|  \| \| \| \| | Control | 104 | 81 | 144 | 74 | 173 | 95 |
|  | Partial | 97 | 88 | 121 | 91 | 138 | 97 |
|  | Full | 116 | 71 | 354 | 81 | 469 | 94 |
| Assesses for perineal and vaginal lacerations | Control | 104 | 91 | 139 | 95 | 169 | 89 |
|  | Partial | 95 | 99 | 120 | 98 | 135 | 99 |
|  | Full | 116 | 90 | 346 | 97 | 462 | 97 |
| Visually assesses for amount of bleeding within 1 min of delivery of placenta | Control | 104 | 50 | 144 | 42 | 173 | 94 |
|  | Partial | 97 | 60 | 121 | 75 | 138 | 96 |
|  | Full | 116 | 61 | 354 | 64 | 469 | 92 |
| Care of Mother (Performed 6+ of 7 tasks) | Control | 104 | 13 | 144 | 11 | 173 | 58 |
|  | Partial | 97 | 36 | 121 | 64 | 138 | 83 |
|  | Full | 116 | 14 | 354 | 37 | 469 | 67 |
| Care of Mother (Continuous, 0 to 7 elements), Mean (Standard Deviation) | Control | Mean  4.2 | (SD)  (1.2) | Mean  4.2 | (SD)  (1.2) | Mean  5.6 | (SD)  (1.2) |
|  | Partial | 4.8 | (1.5) | 5.7 | (1.0) | 6.1 | (1.0) |
|  | Full | 3.8 | (1.4) | 4.9 | (1.4) | 5.8 | (1.2) |
