# Supplementary material for: Peer-assisted learning after onsite, low-dose, high-frequency training and practice on simulators to prevent and treat postpartum hemorrhage and neonatal asphyxia: A pragmatic trial in 12 districts in Uganda
Source: PLoS One. 2018 Dec 17;13(12):e0207909. doi: 10.1371/journal.pone.0207909 (PMC6296740; doi:10.1371/journal.pone.0207909)
Supplement: S2 Table — (DOCX) [file pone.0207909.s002.docx]

**S2 Table. Providers’ Care to the Newborn by Study Group, Direct Clinical Observations**

|  | **Group** | **Baseline**  **(May 2014)** | | **Midline**  **(Jan-Feb 2015)** | | **Endline**  **(Sept-Oct 2015)** | |
| --- | --- | --- | --- | --- | --- | --- | --- |
|  |  | **N** | **%** | **N** | **%** | **N** | **%** |

**Element:**

| Covers baby with dry towel and covers head | Control | 101 | 79 | 139 | 92 | 168 | 97 |
| --- | --- | --- | --- | --- | --- | --- | --- |
|  | Partial | 94 | 89 | 118 | 94 | 136 | 98 |
|  | Full | 114 | 86 | 341 | 95 | 464 | 95 |
| Placed skin-to-skin OR wrapped in a dry towel | Control | 103 | 78 | 140 | 96 | 170 | 99 |
|  | Partial | 96 | 99 | 120 | 98 | 136 | 99 |
|  | Full | 115 | 90 | 344 | 97 | 463 | 97 |
| Visually assesses baby's breathing a second time | Control | 102 | 91 | 136 | 86 | 168 | 98 |
|  | Partial | 96 | 81 | 119 | 98 | 136 | 99 |
|  | Full | 115 | 90 | 341 | 93 | 463 | 96 |
| Encouraged mother to breastfeed w/I 1 hour | Control | 104 | 15 | 144 | 51 | 173 | 75 |
|  | Partial | 97 | 47 | 121 | 86 | 138 | 92 |
|  | Full | 116 | 12 | 354 | 64 | 469 | 75 |
| Care of the newborn – performed 4 of 4 elements | Control | 104 | 13 | 144 | 41 | 173 | 68 |
|  | Partial | 97 | 30 | 121 | 77 | 138 | 88 |
|  | Full | 116 | 9 | 354 | 58 | 469 | 68 |
| Care of the newborn –  (Continuous, 4 of 4 elements), Mean (Standard Deviation) | Control | Mean  2.6 | (SD)  (1.0) | Mean  3.2 | (SD)  (1.0) | Mean  3.6 | (SD)  (0.7) |
|  | Partial | 3.1 | (0.8) | 3.7 | (0.6) | 3.8 | (0.6) |
|  | Full | 2.8 | (0.7) | 3.4 | (0.9) | 3.6 | (0.7) |
