# Supplementary material for: Peer-assisted learning after onsite, low-dose, high-frequency training and practice on simulators to prevent and treat postpartum hemorrhage and neonatal asphyxia: A pragmatic trial in 12 districts in Uganda
Source: PLoS One. 2018 Dec 17;13(12):e0207909. doi: 10.1371/journal.pone.0207909 (PMC6296740; doi:10.1371/journal.pone.0207909)
Supplement: S2 File — (DOCX) [file pone.0207909.s004.docx]

**S2 File. S2 File. CEvans study protocol JHSPH IRB Uganda**

**JHSPH Institutional Review Board**

**RESEARCH PLAN**

| **PI:** | Dr. Cherrie Evans, Jhpiego | | |
| --- | --- | --- | --- |
| **Co-investigators in Uganda:** | Leah Thayer, Jhpiego Uganda  David Serukka, PREFA (Uganda)  Dr. Anthony. K. Mbonye, Ugandan Ministry of Health  Innocent Atukunda, Jhpiego Uganda | **Co-Investigators in Baltimore:** | Dr. Eva Bazant, Jhpiego  Dr. Peter Johnson, Jhpiego  Cyndi Hiner, Jhpiego  Emma Williams, Jhpiego |

**Study Title: Saving Lives at Birth in Uganda: Building and Sustaining Capacity of Frontline Health Workers – A Program Evaluation**

**IRB No.: 5383**

**PI Version Number/Date:** October 16, 2013

1. **Aims/objectives/research question/hypotheses:** Describe the primary and secondary aims/objectives of the research, or the project’s research questions or hypotheses.

**Study Aim:**

The study aims are to evaluate the impact of supportive follow-up strategies for a novel twinned training and capacity-sustaining program among frontline health workers (providers) attending facility-based births in remote and district level health facilities in Uganda. The program is designed to improve provider competencies, provider performance and health outcomes among women giving birth and newborns. The goal is to reduce postpartum hemorrhage (PPH) and birth asphyxia -- leading causes of maternal and newborn mortality. Two curricula will be evaluated: Helping Mothers Survive: Bleeding After Birth (HMS: BAB) and of Helping Babies Breathe (HBB).

The objectives are presented in order of an evaluation logic model: Process, Outputs, Outcomes, and Impact.

**Objectives of the Evaluation:**

***Objective 1 (PROCESS):*** Improve and sustain the frequency of **providers’ practice sessions** each week of key PPH and newborn care interventions.

***Objective 2 (OUTPUTS):*** Improve and sustain **provider competencies** during simulation training related to key PPH and newborn care practices, as measured by the percent of providers who pass, and the mean ‘correct’ score on the knowledge and Objective structured clinical examination (OSCE) assessments of a) HMS:BAB and b) HBB.

***Objective 3 (OUTCOMES):***  Improve and sustain **provider performance** of routine, immediate PPH-prevention and newborn care practices for all mothers/newborns: as measured by the percent of: a) women who received oxytocin or misoprostol within 1 minute of vaginal birth, b) the percent of women among whom the placenta is inspected to standard within 15 minutes, c) the percent of newborns checked for breathing within one minute.

***Objective 4 (OUTCOMES):*** Improve and sustain **case management** of PPH and newborn resuscitation during observations of care, as measured by a) percent of women with undelivered placenta after 30 minutes who are treated with oxytocin, and b) percent PPH cases that receive repeated dose of oxytocin or misoprostol, c) percent of newborns not breathing at birth who are breathing or being ventilated with bag & mask at one minute.

***Objective 5 (IMPACT)***: Reduce over time: a) the percent of facility-based deliveries who experience PPH; b) the PPH-related case fatality rate, and c) the perinatal death rate (the fresh stillbirth rate and early newborn mortality rate at 24 hours or prior to discharge)^[[1]](#footnote-2)^.

Prior to presenting the hypotheses, we describe briefly below the three study arms and three time points of data collection. More description is provided in the Study Procedures section. (See Figure 1, page 11)

**Table 1. Brief Description of Three Study Arms/Groups**

| **Arm** | **Short Name** | **Component 1** | **Component 2** | **Component 3** |
| --- | --- | --- | --- | --- |
|  |  | Training onsite (BAB, HBB) of providers, simulators left at facility | Peer Practice Coordinator (PPC) is trained and expected to hold weekly onsite practice sessions with providers | Mobile messages remind all trained providers to practice scenarios; and PPC has weekly mobile support from district trainer |
| 1 | Training-only | Yes | No | No |
| 2 | Partial Package | Yes | Yes | No |
| 3 | Full Package | Yes | Yes | Yes |

**Hypotheses**

Overall Hypothesis: The partial package intervention (one-time, onsite training of providers, combined with low-dose, high-frequency peer-led practice sessions) and the full-package intervention (adding reminders and mobile support) will lead to significant gains in provider competencies (between immediately post-intervention to midline) and provider performance of key interventions *BAB and in HBB*, which are both maintained once the active intervention ceases.

Specific Hypotheses:

*In BAB and in HBB related to the primary outcomes (see Section 4b5):*

1. Provider competencies and performance will not decline (i.e. will be non-inferior) between midline and endline in the *full* package group.
2. Provider competencies and performance will not decline (i.e. will be non-inferior) between midline and endline in the *partial* package group.
3. At endline, the provider competency level and the performance level of the *partial* package group will be significantly higher than those of the training-only group.
4. At endline, the provider competency level and the performance level of the *full* package group will be significantly higher than those of the training-only group.
5. **Background and rationale:**

***Disease Burden: Postpartum Hemorrhage and Neonatal Apnea/Asphyxia***

Postpartum hemorrhage (PPH) is the leading cause of maternal mortality worldwide (1), accounting for one-quarter of maternal deaths (2). The global incidence of postpartum hemorrhage has been reported at 10.8%, with a range from 7.2% in Oceania to 25.7% in Africa (3). About one-quarter of global neonatal deaths are due to birth asphyxia and another one million stillbirths occur during the intrapartum period—these stillbirths are often misclassifications of secondary apnea. A large proportion of these two million deaths could be averted with the timely and correct use of neonatal resuscitation (4).

Uganda, the setting for this study, has a maternal mortality ratio of 310/100,000 live births (5) and according to regional estimates, about 34% of these deaths are due to postpartum hemorrhage (6). The national neonatal mortality rate is 29 per 1000 live births and the stillbirth rate is 25/1000 (7). It would be expected that 28% of neonatal deaths would be due to asphyxia/apnea in this setting (6). The proportion of births with a skilled attendant is about 80% in urban areas and 40% in rural areas (7).

***Neonatal Asphyxia/Apnea Interventions***

The American Academy of Pediatrics and partners developed “Helping Babies Breathe” (HBB) (8), a training package designed to increase newborn survival by improving recognition and basic interventions for babies who do not breathe at birth. HBB focuses on routine care after healthy birth, initial steps of helping the newborn to breathe at birth, ventilation with bag and mask, and ongoing care after resuscitation, with use of a simulator to practice these skills. Results of HBB field testing in Kenya, Pakistan and India demonstrated improved knowledge and skills in providers and helped refine the educational materials, which are currently in use in 55 countries.

HBB implementation field trials in India and Tanzania have shown improved survival of babies not breathing at birth. In India, in rural primary health centers and district and urban hospitals, Goudar et al found that stillbirth declined from 3.0% to 2.3% (odds ratio [OR] 0.76, 95% confidence interval [CI] 0.59–0.98) and fresh stillbirth from 1.7% to 0.9% (OR 0.54, 95% CI 0.37–0.78) comparing pre and post-HBB training over 5-months; however, early neonatal mortality was unchanged (9). In Tanzania, Msemo et al found a similar reduction in fresh stillbirths in 8 hospitals comparing pre and post-HBB intervention facility data with no control group. HBB was associated with a 24% reduction in fresh stillbirths after 2 years from 155 of 8124 births (19.0 per 1000 to 14.5 per 1000; (RR 0.76; 95% CI 0.64–0.90; p= .001), as well as a 47% reduction in early neonatal mortality within 24 hours (13.4 to 7.1 per 1000 liveborn deliveries, relative risk [RR] 0.53; 95% confidence interval [CI] 0.43–0.65; p= .0001).) (10). A six-country community-based cluster-randomized controlled trial of packaged essential newborn care interventions found no reduction in neonatal or perinatal death but a 31% reduction in stillbirth rate (11). In the same trial, a neonatal resuscitation only intervention resulted in no decline in neonatal or stillbirth rates. Unlike the proposed study and the studies in Tanzania and India, this trial provided no simulators for ongoing routine practice.

Available evidence from Uganda suggests suboptimal coverage and quality of neonatal resuscitation, resulting from a combination of lack of supplies, understaffing of facilities and lack of provider knowledge regarding standardized guidelines (12). HBB is in alignment with Ugandan guidelines and is being scaled up in some districts.

***PPH interventions: Prevention and treatment of PPH***

Active management of the third stage of labor (AMTSL) is critical in reducing the risk of postpartum hemorrhage and has been shown to decrease PPH by as much as 64% (13). Historically, AMTSL consists of 3 interventions: uterotonic within one minute of birth, controlled cord traction (CCT) to deliver the placenta, and uterine massage once the placenta has delivered (14). Based on recent WHO-supported study, uterotonic use within one minute of birth is considered the key component of AMTSL with evidence supporting a reduction in PPH (15). Based on this new evidence, WHO revised the recommendations for PPH prevention focusing on this key component and emphasizing that CCT should be performed by skilled birth attendants only and that checking uterine tone should replace massage. The HMS:BAB curriculum is fully aligned with these new recommendations.

Providers must be trained in AMTSL so that all women receive AMTSL as part of high-quality care on the day of birth. However, it is known that AMTSL is not practiced at high coverage and quality in many developing country settings (14). For example, a nationally representative sample survey in Uganda found that 89% of women delivering in facilities received a uterotonic drug during the third or fourth stage of labor; however, when the practices were evaluated against AMTSL guidelines regarding timing and dosage of uterotonic plus use of controlled cord traction and uterine massage, the study found that only 5-7% of women received AMTSL (16). The materials for our PPH training intervention have been reviewed by WHO and were deemed to be in accordance with the latest guidelines. The HMS:BAB curriculum is fully aligned with Ugandan guidelines.

MCHIP/Jhpiego has carried out observational Quality of Care (QoC) observation studies in several African countries, but not yet in Uganda. In nearby Tanzania, using the strictest definition of AMTSL—oxytocin administered IM within one minute of delivery, controlled cord traction (CCT) and uterine massage following delivery of the placenta—AMTSL was correctly performed in 26% of all deliveries observed (33% of deliveries in regional hospitals, 8% in health centres and dispensaries) (17). The situation was better in Kenya, in the nationally representative QoC study: During observations, oxytocin coverage was 90%, but only 50% of women received AMTSL provided according to standards (18) (Kagame 2012).

***Training and Behavior Change Interventions for Birth Attendants***

Training has been shown to be one important part of behavioral interventions to ensure that providers give a uterotonic during the third stage of labor, along with other cues to maintain behavior change and reinforce training messages (19). In addition to preventing a large proportion of PPH cases through AMTSL, providers need to be trained to recognize and treat postpartum hemorrhage. They also need a mechanism to maintain those competencies, since the actual occurrence of life-threatening hemorrhage can be relatively rare for any single provider, especially those working in setting with low caseload (volume) of births. Simulation-based training, in which simulator devices or models give health professionals an opportunity to practice mechanical and communication skills related to specific scenarios, has been found to result in improvements in clinical (real life) situations (20). *Low-dose, high-frequency (LDHF)* practice has been associated with greater skills retention and transfer to performance. Another promising arena for behavior change is mobile health or mHealth, which involve use of technology. mHealth interventions are increasingly used in developing countries to reinforce training messages and treatment guidelines, to send reminders to providers, and to assess knowledge. For example, Zurovac et al found that brief reminders delivered via mobile phone improved Kenyan health workers’ adherence to malaria treatment guidelines (21).

1. **Participants**:

*Location*

This study will take place in Uganda at Health Centers (level II, III and IV) and District Hospitals, to reach ‘frontline’ health providers who attend births. This study will take place in districts meeting study criteria in two regions to demonstrate ability to implement in different geographic settings and potential for scale up. There are currently 10 regions of Uganda. We reviewed the skilled birth attendance (SBA) rate, under 5 mortality, and average caseload per facility for all regions.

Regional and District selection

These steps were followed in selecting Regions and Districts:

*Step 1.* Of the 10 regions in Uganda, we chose to eliminate the highest and lowest performing. Kampala, Central 1 and Central 2 have the highest ranking maternal and newborn indicators (skilled birth attendance and under 5 mortality) and Karamoja, West Nile and North have the lowest. These government assigned rankings are the most relative to our facility based intervention.

*Step 2*. We surveyed the country with guidance from Ministry of Health publications and consultations, and excluded districts that have or recently had an active maternal health or newborn health intervention, as this would dilute the effect of our intervention or result in high levels of quality of birth care at baseline, with little room for improvement.

*Step 3* We combined Western and Southwest Regions into one group and Eastern and East Central Region into a second group in order to consolidate as Western had only 4 possible districts and East Central had only 3.

*Step 4.* We examined the a) number of facilities by type in each district and b) caseload of births in one year (July 2012- June 2013) in each district, and calculated the average number of births per facility per day. We then selected all districts within the two groups that had an average of > or = to one birth per facility per day. This resulted in 6 districts in the Eastern group and 6 in the Western group.

All public Level II, III, and IV facilities offering birthing services within a district will be included (range of 7-17 per district for a total of 133 facilities). In districts where there is no public hospital, we will include any Private Not for Profit hospital providing birthing services in that district where they exist. In all of these facilities, as mentioned in Section 3c, an estimated 1,300 providers will be included in the study.

**District assignment to intervention arm:**

In each region we will divide the six districts into two groups of three. Within the groups of three we will randomly choose a district name from a hat. For the district pulled we will draw a number card with a “1”, “2”, or “3” from a hat. Those numbers will correspond with the study arms outlined in Table 1 (page 2).

- 1. Describe the study participants and the population from which they will be/were drawn. If you plan to include children, specify their ages and gender.

Study participants include any providers at selected facilities who provide birthing services to women and all women admitted to the labor ward for labor. (Table 2 presents the maximum number of study contacts per participant.

**Table 2. By research objective, Participants (Sample and Population), Data Source, and Number of Contacts**

| **Obj.** | **Study Participants (Sample & Population)** | **Data Source** | **Number of Study Contacts** |
| --- | --- | --- | --- |
| 2 | Facility in-charge or designated provider/pharmacist | Health facility assessment checklist; | Total: 3 (Baseline, Midline, Endline) |
| 2 and 3 | Providers in health facilities who attend births on days and are present on days of training and/or assessment/observation [Population: providers who attend births] | Written knowledge tests and OSCE competency tests for BAB and HBB using simulators | Before and after BAB training  Before and after HBB training day  Midline assessment  Endline assessment  Total: 4 |
|  |  | Direct Clinical Observation (DCO) of births at facility | Total: 3 (Baseline, Midline, Endline); providers may be observed several times over the course of the 2 or 3 day assessments |
| 3, 4 | Women giving birth in health facilities on days of observation, and newborns [Population: women giving birth and their newborns] | Direct Clinical Observation (DCO) of births at facility | 1 (women will likely be observed only with one birth over the period of the study) |
| 4,5 | All women who give birth in study facilities (and newborns) will have their service delivery statistics recorded (without identifiers) and aggregated | Abstraction from Facility registers (existing registers and supplemental information for the HMS:BAB and HBB project) | None |
| 1, 2 | Providers described above who are selected to be Peer Practice Coordinators (PPC) who attend births and are selected to be PPCs due to leadership skills [Population: see above] | PPC practice logs | Weekly, the PPC will record data in the practice log. |
| 1 | Providers, Facility In-charges, and stakeholders such as Ministry of Health in-charges | Semi-structures interviews | 1 |
| 1 | Providers | Focus group discussions | 1 |

- 1. Describe any screening procedures and any **inclusion or exclusion criteria**.

For Providers:

- **Inclusion Criteria**: Health providers who attend births in participating health facility and consent to be assessed at the time of enrollment and at several points in time over two years.
- **Exclusion Criteria:** none

For Peer Practice Coordinators:

- **Inclusion criteria**: criteria for being a PPC include: being an experienced skilled birth attendant and likely to remain at the facility during the study period
- **Exclusion Criteria:** Provider has <1 year of experience, and has plans to be transferred to another facility or leave the facility soon.

For Women in Labor and Delivery and Newborns - Observations of Births

- **Inclusion Criteria**: Women in any stage of labor in participating facility who consent to observation of their delivery and care of their newborn (or consent from the next of kin if the woman is incapacitated and not able to provide consent)
- **Exclusion Criteria**: none

Facility In-charges and Stakeholders

- **Inclusion Criteria**: Facility in-charges at sampled health facilities; stakeholders identified by Jhpiego senior managers as being influential in maternal and newborn health policy decisions in Uganda.
- **Exclusion Criteria**: none

- 1. Provide **sample size** and a **justification** as to how you arrived at your projected sample size.

*Knowledge and Competency Assessments*

For the knowledge and clinical practice assessment, we propose conducting a census of birth attendants working at each facility on the days of training and midline and endline facility data collection. In training interventions, it is routine to test participants before and after the intervention in order to give them feedback to clarify any misunderstandings and improve their performance. We will also do these assessments at baseline and midline facility data collection because we are also interested in collecting data from all providers so that we can do a secondary analysis that will test for a correlation between providers’ performance on knowledge assessments and simulator-based performance tests, participation in practice sessions, and directly observed clinical performance. Table 3 presents the maximum number of providers in the sample facilities, based on data provided by the Ministry of Health; actual enrolled numbers will be lower because not all health care providers on staff will present on the day of data collection. In addition, we recognize there will be overlap between provides at each time point. The outcome measure will be a continuous variable measuring ‘correct’ knowledge and competencies across three OSCE exams of BAB or HBB.

**Table 3. Number of Providers to be Assessed (c)**

| **Region** | **Pre and post BAB training (a)** | **Pre and post HBB Training (a)** | **Midline Assessment (b) (d)** | **Endline Assessment (b) (d)** | **Total Providers in each study group (e)** |
| --- | --- | --- | --- | --- | --- |
| Districts in Eastern | 233 | 233 | 233 | 233 | 932 |
| Districts in Western | 1084 | 1084 | 1084 | 1084 | 4336 |
| **Grand Total** | **1317** | **1317** | **1317** | **1317** | **5268** |

Notes

1. Pre- and post-training, providers will be assessed on knowledge and OSCE competency tests. There will be separate one-day trainings for BAB & HBB spaced 2 months apart.
2. At midline and endline providers will be given BAB and HBB knowledge tests and OSCE skills tests. Facility readiness assessments will also occur at these time points.
3. The number of providers in this table is taken from the ACTUAL number of providers that we are aware exist in the Eastern and Western region that attend births. All providers are invited to participate in the invention and may be assessed.

*Direct Clinical Observation:* Number of births (women and newborns) to be observed

We specify the sample size of number of births to observe below. The outcome variable is: the percentage of women who **received oxytocin** in correct dose immediately after vaginal birth in three study arms. The hypothesized levels of this variable at each time point are presented below:

**Table 4**. Hypothesized levels of “percent of women who **received oxytocin** in correct dose “within 1 minute of vaginal birth” in three study arms

| **Study Arm** | **Baseline** | **Midline** | **Endline** |
| --- | --- | --- | --- |
| Full | 50% (a) | 75% | 70% |
| Partial | 50% | 65% | 60% |
| Training-only | 50% | 55% | 50% |

  Note:

1. A study carried out in 2007 (POPPHI, 2007) found that **20%** of women delivering at health facilities in Uganda received an oxytocin injection between birth and the delivery of the placenta. However, given the small sample size (n=259 births observed) and the fact that the situation may have improved (as it has in many countries), we have conservatively estimated baseline coverage of oxytocin use to be **50%.**

**Table 5**. Number of **births** to observe (women in labor and delivery)

| **Study Arm** | **Baseline** | **Midline** | **Endline** | **Total births** |
| --- | --- | --- | --- | --- |
| **Full Intervention Group** (expected: 50% at baseline to 75% at midline to 70% at endline) | **378**  67 to compare baseline-midline | **378** (a) for midline-endline comparison  99, midline-only comparison  67 to compare baseline-midline | **378** (a) for midline-endline comparison  189 endline-only (b) | **1134** |
| **Partial** **Intervention Group** (expected: 50% at baseline to 65% at midline to 60% at endline) | **189** to compare baseline-midline | **189**, to compare baseline-midline  99, midline-only comparison | **189**, endline-only comparison (b) | **567** |
| **Training-only** **Group** (expected: 50% baseline to 55% at midline to 50% at endline) | **189** | **189**, midline-only comparison | **189,** endline-only comparison (b) | **567** |
| **Grand Total** | **756** | **756** | **756** | **2268** |

 Notes

1. The number of **378 births** is recommended for each time point in the full package group only. N=378 is based on calculations for midline and at endline is based on a (within-group) non-inferiority calculation in the intervention group. We set the number of health facilities at 54 (9 facilities on average per district * 3 districts * each of 2 regions). This sample size will achieve 80% power to detect a non-inferiority margin difference between the group proportions of providing oxytocin of -0.1000. As shown in the previous table, the percent of deliveries with oxytocin provision at midline (post-intervention) is hypothesized to be 70% in Full group. We assume intra-class correlation (ICC) of 0.005 and significance level of the test is 0.0250.
2. The number of **189 births** in each group at endline (only) is based on the following assumptions: This is a two-sample comparison of proportions. We aim to detect a 15% point difference at endline between an intervention group and the training-only group. Training-only group value set at 50%, as this is most conservative. Intervention group value set at 65%. Power is set at 80%. ICC is set at 0.01.
3. To avoid oversampling, and to compensate for different caseloads at different facilities, the study team will be given a target number of births to observe and they will stop when they reach this target.

*Service Statistic Data Abstraction*

There is no sample size calculation for analysis of service delivery statistics because baseline data from the study health facilities are not available.

*Qualitative Data: Focus Group Discussions and In-depth Interviews*

At midline, we will conduct focus group discussions and semi-structured interviews with providers and health facility in-charges, stratifying the sample based on study arm and level of simulator practice sessions achieved, as described in the table below, for a total of 24 health facilities and up to 240 participants. Facilities will be purposively sampled, using data from program evaluation and Jhpiego staff members’ knowledge of contextual factors in the sampled health facilities. Each group of 12 facilities (either high or low adherence) will include different levels of health facilities. Within each facility, all providers that assist at or conduct deliveries will be invited to participate in the focus group discussion. We will also randomly select two providers to participate in a brief, semi-structured interview. The facility in-charge will be invited to participate in a semi-structured interview. Our planned sample size may be higher than needed to reach data saturation. After four facilities have been sampled, the research team will continuously assess notes and transcripts to determine whether data saturation has been reached. Data collection will continue until reaching saturation or until reaching the planned sample size.

**Table 6. – Sample Size for Qualitative Data Collection**

| **Number of Facilities and Providers** | **Facilities with High Adherence**  **to Practice Guidelines** | **Facilities with Low Adherence**  **to Practice Guidelines** | **Total** |
| --- | --- | --- | --- |
| Study Arm 1 | 4 | 4 | 8 |
| Study Arm 2 | 4 | 4 | 8 |
| Study Arm 3 | 4 | 4 | 8 |
|  |  |  |  |
| Total Facilities | 12 | 12 | 24 |
| Total Providers* | 120 | 120 | 240 |
|  |  |  |  |
| Total Focus Group Discussions | 12 | 12 | 24 |
| Total Interviews | 36 | 36 | 72 |
| * Assuming a maximum of 10 providers per facility. | | | |

- 1. Describe whether **identifiers** will be collected.

Women in Labor and Delivery and Newborns - Observations of Births

From women delivering in the facility, only the first name (and in the case of multiple observations, if there are two women with the same first name, the last initial) will be collected *temporarily* from women while they are in the hospital. However, the first names of women will *not* be entered to the database when the data from each tablet are aggregated. There will be no identifiers of women in the electronic database. There will be no identifiers of women written on paper. Other identifiers beyond first name will not be collected from *women* and newborns who are observed.

Service statistics for deliveries and newborn care will be collected from the monthly aggregate reports that do not have the client names (no identifiers).

Providers:

Identifiers will be collected from health providers observed in clinics and/or trained. Provider identifiers will be stored in a database that will be separate from the database storing information about provider knowledge and performance. Rationale: We will follow individual providers over time to track increase in performance or competencies. We need to be able to identify unique providers, such as by collecting name, date of birth, cadre, and health facility, in order to link the data collected on the same provider at various time points (longitudinal analysis). In one study arm providers will be invited to share their mobile telephone number in order to receive content and practice reminders. We will collect no identifiers from providers that participate in focus group discussions.

Facility in-charges and stakeholders:

We will collect no identifiers from those asked to participate in the semi-structured interviews.

Does your study fall within HIPAA requirements because of either of the following:

1) The study team will access, collect, and/or use for research purposes protected health information disclosed by a U.S. health care provider (covered entity), or

2) Identifiable private health information collected elsewhere (including from a foreign country) will be brought to a covered entity here in the U.S. (for example, a JHU SOM laboratory) for analysis?

No. Jhpiego is not a covered entity because it does not provide health care, health insurance or services related to health care billing.

1. **Study procedures:**

**b.** If your study involves contact, direct or indirect, with subjects, provide the following:

1) General **study design and methods**.

The study is a quasi-experimental design with three study arms/groups that receive different levels or intensities and modalities of the program intervention. Figure 1 (page 10) provides an overview of the intervention and evaluation components of the study. Briefly, the same simulator-based training will be provided in all three study arms. After the training, a simulator will be left in the health facility, and providers will be encouraged to practice with it regularly. In two of the study arms, specific health workers will be recruited to support the intervention by encouraging their colleagues to practice with the simulator. In one study arm, the practice will be further reinforced through mobile phone-based support. Following is a more detailed description of each of the three components.

**Component 1 (Training)**: Training is composed of two separate training interventions. First, in each study facility, we will conduct a single day, simulator-driven training on PPH prevention and treatment; all providers who attend births will be invited to participate. Eight weeks later, in each facility, we will conduct a one-day, simulator-driven training for prevention and management of asphyxia in the newborn. After each one-day training, simulators will be left at the facility for practice with a corresponding practice schedule.

**Component 2 (Peer-led Practice Sessions)**: On the day the first training (for PPH), 2 birth attendants at the facility will be selected to serve as peer practice coordinators (PPC). The PPCs will be trained to encourage their coworkers to participate in 15-minute practice sessions each week for 8 weeks, in which they will use the simulators to practice the skills learned in the one-day training. After the newborn asphyxia training occurs, these same PPCs will be trained to support a similar practice schedule for the following 12 weeks - 8 weeks for newborn asphyxia practice and 4 weeks for combined PPH and asphyxia skills practice.

**Component 3 (Mobile phone-based support)**: (Note: This component is being negotiated with the ministry of health in conjunction with UNICEF due to the moratorium on mHealth interventions) Labor ward providers will be given mobile text messages for:

1. Content reminders
2. Practice reminders

PPCs will be connected to the district trainer via mobile phone for weekly phone calls during the practice periods to provide reminders and support for practice.

2) **Study procedures, including sequence and timing.**

Figure 1 presents an overview of activities related to the intervention and its evaluation.

Figure 1 – Intervention and Evaluation Activities

*Intervention Description – More Detailed*

**Intervention: Bleeding After Birth**

The intervention starts with all study arms receiving a one-day, facility based PPH prevention simulator-assisted training of all providers who attend births in a facility with the simulators being left onsite for ongoing practice:

The first module of the twinned training, “Bleeding after Birth” (BAB), uses graphic materials and simulation, to teach providers psychomotor and decision making skills to prevent, detect, and manage PPH including both treatment and timely referral as appropriate. To ensure retention and improved performance, for two of the study arms training will be followed by ongoing **low dose, high frequency (LDHF) simulation practice** conducted by Peer Practice Coordinators at the facility. (The “training-only” study arm will have the PPH simulator left at the facility with a recommended schedule for practice, but no PPCs designated to coordinate the training.) PPCs will be providers already posted at the facility who have undergone the training and have agreed to take on the role to support ongoing training in their facilities. After the initial training module is delivered, two providers per facility will be invited to participate in improving care for PPH and newborns by become PPCs. Two will be selected to address turnover and ensure simulated practice is conducted on a routine basis. After training in the first module (BAB), the PPCs will undergo a half-day training to enhance their skills related to peer-led training.

To test the added value of **mobile phone support**, providers or PPCs in one of the two intervention arms receiving the PPC LDHF intervention will receive ongoing SMS and voice support during the LDHF practice period. PPCs will be provided ongoing mobile support as reminders for practice and to maintain their clinical and training-related capacities. This technology will be used to provide remote reinforcement, assessment and real-time performance support by master trainers to PPCs. In addition, SMS messaging will be used for providers in this intervention arm to provide clinical content and practice reminders.

After a 2-month period of LDHF practice and consolidation of learning, the second one-day training intervention for newborn asphyxia will take place in the facility.

**Intervention: Helping Babies Breathe**

The second module, “Helping Babies Breathe” (HBB), is designed to increase newborn survival by improving recognition and basic interventions for babies who do not breathe at birth. HBB focuses on routine care after healthy birth, initial steps of help to breathe at birth, ventilation with bag and mask, and ongoing care after resuscitation. Again, to ensure retention and improved performance, for two of the study arms (the same two as above) training will be followed by ongoing LDHF simulation practice conducted by the same PPCs at the facility who were trained to provide LDHF practice support after the PPH intervention (the 3rd study arm will have the newborn simulator left at the facility with a recommended schedule for practice.) As above, the intervention arm receiving the mobile phone support for PPH will receive mobile phone support for newborn resuscitation.

During the intervention period, the investigators will work with ministry of health officials to ensure adequate supply of oxytocin, and supplies related to neonatal resuscitation.

**Evaluation Activities:**

Data collected at baseline, midline and endline include: facility assessment, provider knowledge and skills assessment (using a simulator), and direct clinical observation. Baseline data will be collected before the one-day training in BAB. Midline data will be collected after the training and 8-weeks of practice for BAB and HBB, plus the 4 weeks of combined practice. Endline data will be collected six months after the end of the combined practice sessions.

**Times of Data Collection**

- Data will be collected at the facilities (facility readiness assessments and observation of provider performance) at baseline prior to any intervention that involves providers or training. Each visit will take place over 2 or 3 days to a health facility. During this baseline visit time the provider knowledge tests and skills stations (OSCEs) for HMS:BAB and HBB will also be administered.
- On the day of training, data will be collected pre and post-training from training participants. This includes knowledge and OSCEs. BAB and HBB trainings will occur separately at different times.
- Following training and during approximately five months of the active intervention phase:
  - Designated Peer Practice Coordinators (PPC) will log their weekly practice sessions on logs (full and partial arms only).
  - Data on the number of reminders that are sent to providers will be recorded, along with logs of calls between trainers and PPCs (full arm only).
  - Aggregated HMIS data related to PPH, newborn care and mortality data will be collected from routine and supplemental HMIS forms.
- When the five months of the active intervention phase is over, then there will be a midline assessment of providers and facilities: this will again involve a facility assessment, provider knowledge test and OSCEs, observation of provider performance, and qualitative data collection at some facilities. This will take place over 2 or 3 days.
- During the same time period, the research team will contact stakeholders by telephone to schedule interviews with them. Then the interviews will be conducted at the stakeholders’ offices or another location, if requested by the stakeholder.
- Following the midline assessment, there will be a ‘dormant’ phase of no active intervention. This is to determine if competencies and performance is maintained by low-dose high-frequency practice in the absence of donor support. We will return six months later for an endline assessment, which the same as the midline assessment except there will be no qualitative component.

**Table 7. – Description of Data Collection Instruments**

| **Tool #** | **Title** | **Content** |
| --- | --- | --- |
| 1 | Health Worker Listing | Name and cadre for each health worker at each facility. This will be used to generate a unique ID for each provider |
| 2 | Facility Inventory | Information about the tools and equipment available at the facility related to the study intervention, such as the availability of uterotonic drugs and neonatal resuscitators. |
| 3 | Direct clinical observation tool | Structured checklist that observers will use to take notes on the care provided to women and neonates, including routine care and treatment for postpartum hemorrhage or neonatal asphyxia. |
| 4 | Supplemental maternity register | ***Note:*** *We will provide this register to facilities, but Jhpiego researchers will not abstract individual records from this document (facility staff will do this).* This register collects data about each birth that is relevant to this study, such as whether a women was diagnosed with postpartum hemorrhage and whether a newborn received bag & mask resuscitation. |
| 5 | Supplemental monthly summary report | This aggregated monthly form collects facility-level indicators related to this study, such as the number of women diagnosed with postpartum hemorrhage and the proportion of those women treated with oxytocin or misoprostol. |
| 6 | Participant Characteristics – Baseline | This self-administered questionnaire collects information about qualifications of training participants and their prior experience and confidence related to preventing and treating postpartum hemorrhage and neonatal asphyxia. |
| 7 | Knowledge Test: Bleeding after Birth | 15 multiple choice questions that test providers’ knowledge of the content taught in the Bleeding After Birth training. |
| 8a-c | Observed Structured Clinical Examinations (OSCEs): Bleeding After Birth | Structured checklists that trainers will use to examine providers’ skills related to routine care for third stage labor, retained placenta, and severe hemorrhage due to atony. Providers will demonstrate skills on a simulator. |
| 9 | Peer Practice Coordinators’ Practice Log | In two of the study arms, peer practice coordinators will use this form to record when the providers at their facility practice with the simulator and the topic that they practice. |
| 10 | Phone Support for Peer Practice Coordinators | This will log the data and content of the weekly phone support that trainers have with peer practice coordinators (in one study arm only.) |
| 11 | Opt-in Sheet for Mobile Phone Reminders for Providers and PPCs | In one study arm, providers will have the option of receiving reminders about training content via their personal mobile phones. |
| 12 | Knowledge Test: Helping Babies Breathe | 17 multiple choice questions related to the content taught in the Helping Babies Breathe training. |
| 13 a, b | OSCEs: Helping Babies Breathe | Structured checklists that trainers will use to examine providers’ skills related to neonatal resuscitation using stimulation and ventilation equipment. Providers will demonstrate skills on a simulator. |
|  | *Qualitative* |  |
| 14 | Focus group discussion guide | Health care providers will be asked about their experiences with the program, particularly what the factors that motivated or discouraged them from practicing with the simulator. |
| 15 | Semi-structured interview guide: Health facility in-charges | Health facility in-charges will be asked about their experiences implementing the program and for their opinions about how to strengthen and sustain it. |
| 16 | Semi-structured interview guide: Health care providers | Health care providers will be asked about their experiences with the program and their ideas for how to improve care for postpartum hemorrhage and neonatal asphyxia. |
| 17 | Semi-structured interview guide: Stakeholders | Stakeholder will be asked about their opinions about how to strengthen and sustain this program and other efforts to improve care for postpartum hemorrhage and neonatal asphyxia in Uganda. |

3) *Number of study contacts* or visits required of participants.

Please see table 2 on page 5 for details. Briefly, women in labor, key stakeholders and facility in-charges will each have a maximum of only one study contact. Providers could have up to 12 study contacts, if they were selected for all types of data collection, but this will be rare. Similarly, peer practice coordinators could have up to 12 study contacts plus regular telephone contact with a member of the research team.

4) Expected **duration of the study**.

After JHSPH IRB and Ugandan IRB are obtained, the study will take 18 months.

5) A brief **data analysis plan** and description of the **nature of the variables** to be derived.

**Primary Outcome:**

- For the postpartum hemorrhage component, the primary outcome will be the proportion of women who received **oxytocin** (at correct dosage) within one minute of a vaginal birth, before delivery of the placenta. Data for this outcome will be obtained from the direct clinical observations.
- For the neonatal asphyxia component, the primary outcome will be the proportion of babies not breathing at birth who are breathing or being ventilated by one minute.

**Secondary Outcomes:**

- Change in providers’ knowledge and competence related to postpartum hemorrhage and neonatal resuscitation, as assessed by knowledge tests and observed performance with a simulator.
- Trends in PPH and neonatal asphyxia/mortality rates, by abstracting *aggregated* monthly service statistics from each facility.
- Identification of structural, environmental and behavior factors that are associated with routine, frequent practice with the simulator.

Data will be entered using Ms Excel/Foxpro/SPSS/CsPro and analyzed using Stata 12.

*Quantitative data analysis*

Specific Hypotheses:

*In BAB and in HBB related to the primary outcomes (see Section 4b5):*

1. Provider competencies and performance will not decline (i.e. will be non-inferior) between midline and endline in the *full* package group.
2. Provider competencies and performance will not decline (i.e. will be non-inferior) between midline and endline in the *partial* package group.
3. At endline, the provider competency level and the performance level of the *partial* package group will be significantly higher than those of the training-only group.
4. At endline, the provider competency level and the performance level of the *full* package group will be significantly higher than those of the training-only group.

Hypotheses 1 and 2:

This is a non-inferiority analysis. Two-sided 90% confidence intervals (CI) for the difference in average knowledge and competence scores between midline and endline will be constructed, separately for full and partial package groups. Non-inferiority will be determined comparing the upper bound of the 90%CI for the difference between the 2 time points to the pre-specified non-inferiority margin of 0.06 (22). If the CI's upper bound is less than the predefined margin, then we are able to claim non-inferiority of the endline knowledge scores to the midline knowledge at a 0.05 level of significance.

We will first examine the differences in sample means of knowledge scores between midline and endline not adjusted for any potential confounding variables. The confidence intervals for the knowledge scores will be calculated using generalized linear models with knowledge and competencies as the dependent variables and time variable (endline vs. midline) as the primary independent variables. To account for repeated observations within providers at midline vs. endline, we will use robust Huber/White estimate of variance implemented in STATA through ‘vce’ (cluster) option. In addition, adjusted 90% CI for the difference in means will be constructed by adding provider- and facility-level factors in the model.

Hypotheses 3 and 4:

We will examine differences in competencies and performance at endline between the 3 study groups using generalized linear models. The dependent variables in these models will be:

1) knowledge and competency scores,

2) proportion of women who received **oxytocin** (at correct dosage) within one minute of a vaginal birth, 3) proportion of women whose placenta was inspected within 16 minutes, and

4) proportion of newborn checked for breathing within 1 minute.

The primary independent variable will be the study group represented as 2 indicator variables for full and partial training package with training-only as the reference group. For these hypotheses, we will only use endline data. Additional variables, related to provider- and facility characteristics predictive of these outcomes, such as cadre, number of years of provider’s experience, and number of prior births attended in last 90 days, will be also included in the model. Analysis of residuals will inform whether the assumptions of the generalized linear model, such as normality and homoscedasticity of residuals as well as linear fit, are satisfied.

Additional analyses will include:

1. At baseline, midline and endline we will collect data from **all study facilities** consisting of service delivery statistics, and facility inventory. These data will be analyzed using population-average marginal generalized linear models estimated using generalized estimating equations (GEE) with exchangeable working correlation structure to account for repeated observations within facilities. The dependent variable will be service delivery statistics. The independent variables will include time (represented as 2 indicator variables for midline and endline with baseline as the reference group), study group (represented as 2 indicator variables for full and partial training groups with training-only as the reference group), and interaction terms (study group by time). The interaction terms will be examined to assess whether there are differences between study groups that change significantly over time.

2. Providers who are trained will keep **logs of their practice sessions.** This is process evaluation data. These data (for example on number of times a scenario is practiced) will be compared across the three study groups using generalized linear models with Poisson or Negative Binomial distribution for the outcome.

3. **Routinely collected data from health facility registers** (recorded daily but aggregated monthly) on services provided to clients and health outcomes (discharge, or deaths and stillbirths) will be analyzed using longitudinal data analysis to account for repeated observations at each facility. This will be for population-averaged marginal generalized linear models using generalized estimating equations (GEE) and exchangeable working correlation structure.

*Qualitative Analysis*

We will conduct a doer/non-doer analysis that will contrast providers and managers at facilities that adhere to low-dose/high-frequency practice recommendations with providers at facilities with few or no reported practice sessions. We will also analyze interviews with key stakeholders to identify their recommendations regarding program sustainability. Audio-recordings of focus group discussion and interviews will be transcribed and entered into Atlas.ti software. Data from close-ended questions will be entered into Microsoft Excel or Stata. Pre-determined and emergent codes will be developed, and content analysis will be conducted.

6) If human biospecimens (blood, urine, saliva, etc.) will be collected . **N/A**

7) Describe how **subjects** will be **screened for eligibility and assigned** to study/intervention and comparison/control groups.

All providers who attend births at the facilities will be invited to participate. All women who are in labor at the facility on the days of observation will be invited to participate (delivery and newborn care will be observed).

Criteria for being a PPC include: being an experienced skilled birth attendant and likely to remain at the facility during the study period (i.e. no imminent transfers).

8) Explain and justify whether there will be **blinding**.

Randomization or blinding of individual subjects is not feasible in this study because of the district-randomized design.

9) Explain and justify whether participants **will not receive routine care** or will have current therapy stopped.

Women in labor and delivery (participants) and their newborns will receive routine care as per Ugandan guidelines.

10) Explain and justify the use of a placebo or **non-treatment group**.

All study groups will receive the training on Bleeding after Birth and Helping Babies Breathe, and simulators (anatomic models) will be left behind at all facilities. The training-only (comparison) group will be encouraged to practice regularly but will receive no peer support intervention or SMS messages.

BAB and HBB are known to improve providers’ knowledge and skills immediately after training (Ersdal 2013, Evans 2013 (in press). The question is how to ensure continued improvement or maintenance in competency and practices outside the clinical trial setting, in peripheral health facilities that are not typically ‘study sites.’

This study will yield the evidence for efficacy of the low, medium, high intensity continuity package. Based on the results, we will make recommendations to the government to inform feasibility of scale-up.

11) Provide a definition of treatment failure or **participant removal criteria**.

Not applicable because we are providing no treatment.

12) Describe what happens to participants receiving therapy when the study ends or if a subject’s participation ends prematurely.

This study is not providing any novel therapy for patients. For providers who are participants, simulators will remain at ALL facilities after the study ends, enabling the providers to continue practice sessions if they want to do so. For women and newborns, this study is not providing any therapy outside of routine care for PPH and newborn asphyxia.

13) Describe the process for **referring subjects** to care outside the study, if needed.

We are providing no treatment as part of this study. However, our training to providers will emphasize the existing referral mechanisms for women who experience labor complications. Each facility will be asked to identify their referral plan. Facility staff will be asked to post on the wall their local transportation plan for referrals from the facility to a higher-level facility.

14) For studies that evaluate interventions, have a randomized study design, and/or are a clinical trial, **provide power calculations** for projected sample size.

Please see section on sample size.

1. If you will perform diagnostic tests, provide the following… **N/A**

**5. Data Security and Protection of Subject Confidentiality (NOTE: LOSS OR THEFT OF COMPUTER OR HARD COPIES OF DATA COLLECTION SHEETS DURING TRANSPORT IS GREATEST THREAT TO SUBJECT CONFIDENTIALITY – BE SURE TO TRAIN YOUR STAFF ABOUT THIS PROBLEM.)**

1. Will the study data stored in the United States be protected by Certificate of Confidentiality? NO.
2. Identify the data security plan below that best describes how you will minimize the risk of a breach of confidentiality by typing an X in the appropriate box on the left side of each section (A, B, C) of this chart. If your study includes sequential phases that require different procedures, or does not fit these categories, explain in “Other”. These categories reflect minimal standards; you may impose more stringent protections. See the JHSPH Data Security Guidance at <http://www.jhsph.edu/irb/Guidance_and_Policies.html>.

| *Note: Identifiers include direct identifiers such as name, address, SSN, hospital record number, etc., and other indirect identifiers (e.g., date of birth, tribe) that, when combined with other variables, may make a subject identifiable. It is possible that a unique, randomly-assigned, study identifier may remain within a dataset, but the dataset could be considered sufficiently ‘deidentified’ for the purposes of the JHSPH IRB. This may be the case if the person in possession of the data cannot use the unique identifiers to locate or identify a specific individual without additional codes or identity table linkages.* |
| --- |

| 1. **Hard (Paper-based) copies of data collection forms:** | |
| --- | --- |
|  | The study collects data that are anonymous; no personal identifiers are recorded or retained from any study participants in either direct or coded form. |
|  | Hard copies of data collection materials have identifiers and are locked in a secure cabinet or room with limited access by specified individuals. COPIES WILL BE KEPT IN INVESTIGATOR’S POSSESSION DURING TRANSPORT. When possible, redacted (de-identified) versions of the data collection sheets will be used for coding and analysis. |
|  | Hard copies of data collection materials include an ID code but do not have personal identifiers. However, a code linking the data to the subject’s personal information is stored separately from the data collection sheets, and is either stored in a secure electronic database, and/or locked in a secure cabinet or room with limited access by authorized individuals. CODE WILL BE KEPT IN INVESTIGATOR’S POSSESSION DURING TRANSPORT. |
|  | Data are not collected on paper. |
| x | Other (describe):  Providers’ identifiers will be collected but not kept with the study data (kept separate) for all data sources except the logbooks of provider practice, log of telephone contacts between trainers and peer practice coordinators, and the list of names and telephone numbers of providers that opt in for receiving SMS messages related to the training. Logbooks that record practice sessions that will include health care providers’ names will be kept in the simulator case in the possession of the PPCs. For all other data sources, hard copies of data collection materials will be kept locked in a secure cabinet or room with limited access by specified individuals. COPIES WILL BE KEPT IN INVESTIGATOR’S POSSESSION DURING TRANSPORT. When possible, redacted (de-identified) versions of the data collection sheets will be used for coding and analysis.  Women’s identifiers will not be collected on paper at all. |
| 1. **Electronic Databases:**   *Note: This refers to the initial database into which study data is entered and stored. If this “Study Database” includes personal identifiers from participants, only de-identified analytic datasets should be used for data analysis except in instances in which identifying information is required.* ***Databases that retain identifying information require a higher degree of electronic security.*** | |
|  | The study collects data that are anonymous; no personal identifiers will be recorded or retained from any study participants in either direct or coded form. |
|  | Personal identifiers are included in the database. If breach of confidentiality poses more than minimal risk to participants because data are personally sensitive in nature (for example, involve substance abuse, mental health, genetic propensities, sexual practices or activities), access to identifiers will be restricted. These data are stored on a secure server protected by strong password, and will be only accessible by authorized study personnel. Data will be coded when possible. Identifiable data transferred or stored via portable electronic devices (e.g., laptops, flashdrives) will be encrypted. The devices on which this information is stored are accessible only to individuals who need access to these data. |
| X | Other (describe):   - Provider identifiers will be collected and will be kept in a separate ELECTRONIC DATABASE from the study data, - The electronic database with the providers’ data (on competency and performance) will include only the unique ID numbers of providers and no identifiers.   These data are stored on a secure server protected by strong password, and will be only accessible by authorized study personnel. Data will be coded when possible. Identifiable data transferred or stored via portable electronic devices (e.g., laptops, flashdrives) will be encrypted. The devices on which this information is stored are accessible only to individuals who need access to these data.  Women’s first name only (and in the case of multiple women being observed and two women have the same first name, the initial of the last name) will be collected *temporarily* on tablets when the data on births are being observed in the health facility; however, once data are transferred to the electronic database the women’s first name and last initial will be deleted. The electronic database will not have women’s identifiers. |
| 1. **Analytic Datasets:**   *Note: This refers to the use, for analysis, of either discrete subsets or the entirety of the database into which study data is entered and stored. To the extent possible, analytic datasets should be de-identified, except in instances in which identifying information is required.* ***Analytic datasets that retain identifying information require a higher degree of electronic security.*** | |
|  | The study collects data that is anonymous; no personal identifiers will be recorded or retained from any study participants. |
| X | Electronic database will be managed by a specific data administrator (PI or other designated person) who will track and log issuance of analytic datasets, and return/removal when approved use ends. Access to analytic datasets will be subject to conditions established by the PI. Electronic analytic datasets will be provided to authorized study personnel, or approved investigators outside the study, with the same data protection requirements established for the study database. |
|  | Other (describe): |

1. If you are using participants’ personal identifiers, describe any plans for **disposing of identifiers** including if, when and how that will be done.

Provider identifiers will be disposed of 1 year after the project end date.

d. Describe any plans for **destroying data** including if, when and how that will be done. JHU policy states that the PI is the steward of research data and is responsible for all information (recorded on any media) and materials (biological or environmental samples) (see: http://jhuresearch.jhu.edu/Data_Management_Policy.pdf.) This responsibility includes physical custody and/or control of the data, storage and sharing (with data use agreements).

Hard copies of data and electronic recordings of focus group discussions and interviews will be disposed of 1 year after the project end date. Electronic databases without any provider identifiers will remain and will not be destroyed, for possible future additional analyses.

**6. Recruitment process:**

- 1. Describe how, and from where, **participants will be recruited**.

All participating providers offering services on the day of birth approved by the facility in charge will be invited to participate in the study. Some providers will be identified by the district authorities and invited to the training on Bleeding After Birth and Helping Babies Breathe. Other providers may learn from those trained providers and be observed by our study.

Peer practice coordinators will be identified by the facility in-charges and district officials from each participating district, and the providers identified will be invited to participate as a PPC. They will receive special training for their PPC role.

Women in labor at the facilities on the days of observation will all be invited to participate. We will approach them in the facility.

Stakeholders will be identified based on the knowledge of Jhpiego/Uganda leadership and in consultation with the Uganda Ministry of Health. They will be contacted at their place of work in person and asked to participate in the study.

- 1. Explain how your **recruitment materials** will be used.

No written recruitment materials will be used. Both health care workers and pregnant women will be recruited orally, immediately before being invited to participate. No recruitment script will be used; the oral consent script will be read to inform potential participants about the study.

- 1. If relevant, address any **privacy concerns** associated with the recruitment process.

We will seek to ensure privacy for providers and pregnant women by obtaining informed consent in a private room or area.

**7. Consent process and documentation:**

1. If you will obtain informed consent from participants, identify the countries where the research will take place and the languages into which each consent document will be translated. If the language is unwritten, provide information about how you will ensure accurate and informed translation, including possible use of audio recording.

| **Country** | **Consent Document**  **(Indicate “All”, or specify each document when translations vary)** | **Languages** |
| --- | --- | --- |
| Uganda | Oral Consent #1 (Health Care Providers and PPCs) | English |
|  | Oral Consent #2 (Pregnant Women) | English and Luganda |
|  | Oral Consent #3 (Stakeholders and Facility In-charges) | English |

1. Describe **who will obtain informed consent** from participants, and how, when and where consent will be obtained. If you include children, be sure that consent is obtained from the person who has the legal authority to provide informed consent. In the U.S., that person is usually a biological parent unless a legal proceeding intervenes. Address this issue for **international studies**. Note: Children in foster care may not be enrolled unless investigators request their inclusion and explain and follow local requirements.

All data collectors will be trained in depth on how to obtain informed consent during a one-week data collector training workshop using the, **“**JHSPH Human Subjects Research Ethics Field Training Guide.**”**

The research team will obtain permission from the district authorities and the facility in-charge prior to collecting data at facilities. Verbal informed consent will be requested from health care providers including PPCs at each data collection point – baseline, before each training, midline and endline. At baseline, midline and endline, data collectors will seek informed consent in a private setting such as a closed office or meeting room. Before trainings, the informed consent script will be administered in a group setting, when the participants have gathered in the training room. At subsequent data collection points, individual informed consent will be obtained with each participating provider.

For the direct clinical observations, we will request informed consent from pregnant women (or if she is unable to give consent during labor and delivery, her next of kin); this consent will also serve as a parental permission form to observe treatment provided to the newborn. The age of majority is 18 in Uganda, and pregnant women less than 18 years old are considered emancipated minors, able to provide consent for themselves. In addition, women may come in with obstetric complications that render them unconscious or semi-conscious and unable to provide consent. Because these cases are very important in our assessment of quality of care, we propose to ask the next of kin to provide consent for incapacitated pregnant women.

1. If the study will involve **vulnerable populations** (e.g., children, prisoners, cognitively impaired adults, non-English-speakers, etc.) describe efforts to ensure their understanding of the research and the **extra protections** that will be in place to ensure their voluntary participation. Include a description of your assent process for children of appropriate age and maturity; and for adults who lack capacity to provide informed consent.

The consent form will be written in simple language and read aloud in the language commonly used, Luganda, in the study area to ensure that it is understood.

The oral consent process will be followed so that all women in labor understand the study participation is voluntary and can be terminated at any time without reason and without penalty. We will ensure that their participation is voluntary. Women will be ensured that they will receive the routine services (delivery and newborn care), whether or not she is a study participant. Women will be asked if they have any questions in their local language and be answered so they fully understand.

1. If a waiver of consent or a **waiver or** alteration **of signed consen**t is requested, provide a justification for the waiver/alteration, and describe any alternate procedures for informing participants about the research.

For health care providers and pregnant women, we request a waiver of signed consent, which is justified by the following conditions:

- The study is low risk.
- A waiver will not adversely affect the rights or welfare of recipients.
- In this setting, written consent for pregnant women is inappropriate, because many women have low literacy levels

**8. Risks:**

- 1. Describe the risks associated with the study and its procedures, including physical, psychological, emotional, social, legal, or economic risks.

| **Risk** | **Description** | **Mitigation Strategy** |
| --- | --- | --- |
| Physical | There are no physical risks to providers or women in labor and delivery from this study. | n/a |
| Psychological/ Emotional | There are minimal psychological/emotional risks, only possible discomfort with being observed or possible concern that the data will not be kept confidential. | The observers and study team will clearly explain the purpose of the assessment to the district authorities and facility-in-charges and obtain their approval for entering the facilities. The observers will explain the purpose of the assessment to **providers and women**, and obtain informed consent from **providers and women** in labor. Observers will be external to the facility. They will answer any questions. In addition, it will be explained to them that their observer is a trained maternity care provider. Observers will be as unobtrusive as possible. A strategy for keeping data confidential will be developed, followed and explained. |
| Social, legal or economic | There is a risk to providers of possible loss of confidentiality of knowledge, test scores, and practice sessions and observation data on the performance of quality of care. | Information on the providers’ performance on assessments or during direct observation of care will not be shared with their supervisors. Provider names will not be recorded on the data collection forms for assessments or observations and their identities will be kept separate from their data.  Providers’ practice sessions with fellow providers will be logged using their names so PPCs can easily see who has practiced in a given week and whom they need to invite to practice. This log will be kept in a folder in the simulator case and will not be left out for casual review. In addition, supervisors will be told that provider refusal to participate in the study or practice sessions should in no way impact provider employment.  To ensure that the observations will not affect provider’s employment, we will only provide aggregate data to facility supervisors and District staff. We will keep single-provider performance information confidential. We will develop a data flow and confidentiality plan.  Results for individual facilities may be reported to the MOH if requested, but reports for external dissemination will not present data at a level lower than a district.  There is no social, legal or economic risk to women. |
| If sub-standard (actual) care is observed and deemed dangerous by observers | There is a possibility that the actual care provided to the woman in labor or delivery or the newborn is sub-standard and dangerous for the mother or babies’ survival (related to reality of the situation, not the observation). | Where available, a senior provider (physician or midwife) will be notified that on the day of assessment, the senior provider must be present in the facility or immediately reachable and available in order to be called in for assistance in cases where patients’ health status or safety is compromised and is not being appropriately managed by the observed provider. This requirement will be outlined in a letter from the MoH and in country PI that will be sent to the director of selected facilities that will inform them that their facility has been selected for assessment in the survey, describing the assessment and activities in the facility and requesting their cooperation. It will also outline the requirement that on the date of assessment where available, a senior provider must be made aware that they may be called in to intervene and should be onsite or immediately available. If a second provider is not available, the study observer who is a senior clinician can choose to intervene.  This condition may intensify the potential Hawthorne effect of being observed - where the observed provider changes their behavior in response to being observed. Knowledge that another [senior] staff member could be called to assist in case of a complication may intensify this effect, such that quality of care observed may be better than routinely provided, but this potential effect is acceptable given the necessity to ensure optimal patient care and safety. |

- 1. Describe the **anticipated frequency and severity of the harms** associated with the risks identified in 8.a., above; for example, if you are performing “x” test/assessment, or dispensing “y” drug, how often do you expect an “anticipated” adverse reaction to occur in a study participant, and how severe do you expect that reaction to be?

We expect any harm associated with the above risks to be rare. Since we are providing no treatment as part of this study, adverse reactions would not be expected.

- 1. Describe steps to be taken to **minimize those risks**.

See above table, column 3.

- 1. Describe the **research burden** for participants, including time, inconvenience, out-of pocket costs, etc.

| **Data Source** | **Research Burden** |
| --- | --- |
| Health facility assessments |  |
| Direct clinical observation (DCO) | Observations of care during labor and delivery and the immediate newborn period will not entail a research burden. No additional time of the providers or women is involved, except for the time needed to explain the study and obtain informed consent. |
| Knowledge and competency assessment (OSCE) of providers | This will take 60 minutes. Pre- and post-training assessments are standard procedure during training workshops, and are expected by providers who participate in training. They will also be collected at midline and endline. |
| Peer practice coordinator (PPC) weekly logs | This may involve a 30 minute burden per week. |
| Abstraction from Facility registers (existing registers and supplemental information for the HMS:BAB and HBB project) | Health facility registers will be modified to collect data related to post-partum hemorrhage and newborn care. The registers will record data on routine services that are to be provided to all mothers and newborns. Registers may also collect data on the care provided to cases, to clients with PPH and newborn asphyxia. There may be separate registers for deaths. Any modifications to standard registers or supplemental registers will be approved by MOH and district authorities. Participating providers may spend an extra 30 minutes per week documenting services provided and outcomes of the patients. |
| Focus group discussions | Up to 1 hour |
| Semi-structured interviews | Up to 30 minutes |

- 1. Describe how participant **privacy will be protected** during data collection if sensitive questions are included in interviews.

This study does not collect sensitive information. Observations will occur where services are normally offered to women in labor and delivery wards. Observers will be as unobtrusive as possible.

During pre and post-training assessments, providers will be assessed in private, in a way that other providers cannot see or hear the simulations, and data when assessments are taken will be kept confidential.

Registers on services provided to patients will be kept as they normally are in the facilities.

**9. Benefits:**

1. Describe any **potential direct benefits to participants** from participating in the research (not including payment for participation)

Benefits to Providers

All **providers** participating will receive basic training in Bleeding After Birth and Helping Babies Breathe. This will likely improve care, at least in the short run, and possibly improve health outcomes. If the SMS component has an effect and improves providers’ skills and services, then providers and clients may benefit. If the low-dose high-frequency training intervention improves providers’ skills, facility readiness, and care to mothers and newborns, this will benefit providers and clients’ health outcomes. This is what we are studying.

The facility directors and staff will be informed that the intervention may benefit participating facilities, staff and clients, or it may not. The results of the study will be disseminated to stakeholders with the intent of improving quality of maternal and newborn care in all facilities in the country.

Benefits to Women Observed

During the planned observations of L&D care, due to the Hawthorne effect of observation, providers may provide better quality of care to clients during L&D that are observed. As discussed, if a patient’s care is observed as substandard to the point of any danger to her or her infant, our strategy to call for additional support from another health care provider in the facility may provide direct benefit to these patients.

1. Describe **potential societal benefits** likely to derive from the research.

The information collected is intended to be used to design interventions and policies to promote more effective delivery of quality MNC health services in the study country. Future clients will benefit in future from improved maternal and newborn care services in the country if study findings stimulate programmatic and policy change.

1. **Payment:**
   1. Describe the form, amount, and schedule of payment to participants. Reimbursement for travel or other expenses is not “payment,” and if the study will reimburse, explain.

No participants will receive payment or reimbursement. No mobile phones or SIM cards will be given to providers in the full intervention study arm.

- 1. Include the possible total remuneration and any consequences for not completing all phases of the research. **N/A**

1. **Drug Products, Vitamins, Dietary Supplements and Devices: N/A**
2. **Safety monitoring:**
   1. Describe how participant safety will be monitored, by whom, and how often.

Services will be provided to clients as part of routine MOH service provision. No adverse events as a result of this study are anticipated. If complications arise during routine service provision, the regular mechanisms will be followed, such as contacting senior providers and initiating referrals. (See Section 4b13).

- 1. If a Data Safety Monitoring Board (DSMB), or equivalent. will be established, describe the following: N/A

i. The DSMB membership, affiliation and expertise.

ii. The charge or charter to the DSMB.

iii. Plans for providing DSMB reports to the IRB.

- 1. Describe plans for interim analysis and stopping rules.

1. **Plan for reporting unanticipated problems/adverse events**:

Describe plan for reporting to the IRB and (if applicable) to the sponsor. Include plan for government-mandated reporting of abuse or illegal activity.

We do not expect any study-related adverse events. See section 12 and 4b13.

Should any adverse event occur in relation to or at the time of the data collection, the data collectors will be trained to notify their team leader immediately by phone, who will notify the co-investigator, Leah Thayer, the Jhpiego Uganda Country Director. Leah Thayer will notify the PI immediately.

If the adverse event is related to the health of the participant, trainers or data collectors will work the health workers to ensure that the participant is attended to as soon as possible, inform the nearest health facility by phone and organize transport for participant and accompanying relative promptly. The trainers or data collectors will inform the facility in-charge. At the same time, the team leader will inform Leah Thayer who in turn will liaise with the DCMOs. Leah Thayer will consult with the PI. The PI will report any adverse event to the IRB within 3 business days.

1. **Other IRBs/Ethics Review Boards:**

This study will be reviewed by the institutional review board of the Makerere University School of Public Health in Kampala, Uganda (FWA#00011353).

1. **Outside collaborations:**

The American Academy of Pediatrics is collaborating with Jhpiego to provide the training and mentoring in immediate newborn care and resuscitation, HBB. They will also provide input on newborn indicators, analysis, and any other newborn related instruments.

Protecting Families Against Aids (PREFA) will hire and manage data collectors for the study with close involvement and oversight by Jhpiego for project management. Our goal is to build the capacity of PREFA for MNH work and for data collection. Jhpiego will adequately train PREFA staff and the data collectors in ethical conduct of research. PREFA will also leverage their relationships within districts in order to introduce the project to the District Health Officers.

**Roles and Responsibilities Matrix for IRB Application**

**Insert Institutions in Collaborator column(s); add additional columns if necessary.**

|  | Jhpiego | Collaborator 1  PREFA | Collaborator 2  AAP |
| --- | --- | --- | --- |
| Primary Grant Recipient | Jhpiego |  |  |
| Subcontractor |  | Prefa | AAP |

**For the following, indicate “P” for “Primary”, “S” for “Secondary” as appropriate to role and level of responsibility.) Add additional items if useful.**

| 1 | Human subjects research ethics training for data collectors | P | S | S |
| --- | --- | --- | --- | --- |
| 2 | Day to day management and supervision of data collection | P | S |  |
| 3 | Reporting unanticipated problems to the JHSPH IRB/Sponsor | P | S | S |
| 4 | Hiring/supervising people obtaining informed consent and/or collecting data | P | S |  |
| 5 | Execution of plan for data security/protection of participant data confidentiality, as described in Sect. 5. | P | S | S |
| 6 | Biospecimen processing, storage, management, access, and/or future use | n/a |  |  |

1. **Oversight plan for student studies: Not applicable**
2. **Oversight plan for studies conducted at non-JHSPH sites, including international venues, for which the JHSPH investigator is the responsible PI:**

The study will be managed by Dr. Evans with assistance from Jhpiego and Prefa staff in Uganda. Dr. Evans and other co-investigators based in Baltimore will travel to Uganda several times for the preparation of data collection, training of assessors, and the supervision of the initial data collection and to oversee program implementation and data collection. The PI or a co-investigator will be present or in daily email contact during the training of district trainers. When the PI is not in Uganda, she will hold regular email and phone communication with the study team, no less than monthly. To ensure a common understanding of the IRB-approved study protocol, each staff person who will be working on the study will be given a copy of the study protocol and will be asked to initial each page to signify that they have read and understand the protocol. A special training will take place where each page of the study protocol is understood.

| **Name** | **Role** | **Institution** | **Background** | **Study Responsibilities** |
| --- | --- | --- | --- | --- |
| Cherrie Evans, CNM, DrPH | Principal Investigator | Jhpiego | Dr. Evans is a clinician and researcher with over 20 years of experience in maternal health including implementation research. | Dr. Evans is based in Baltimore and will provide oversight of the project and oversee the general implementation of the study. |
| Eva Bazant, DrPH, MPH | Co-investigator | Jhpiego | Dr. Bazant is a program evaluator and health services researcher with 15 years of experience in international maternal and reproductive health, and has a focus on evaluation of standards-based quality improvement and provider performance in Sub-Saharan Africa. | Dr. Bazant is based in Baltimore and provides technical assistance related to the study design and data collection, management, and analysis. |
| Leah Thayer | Co-investigator | Jhpiego | Ms. Thayer brings 15 years of experience supporting and managing health programs in African countries; and has cultivated an intimate knowledge of Ugandan institutions and the health system within her 12 years in Uganda. | As country director of Jhpiego in Uganda, Ms. Thayer will supervise the program when Dr. Evans is not present. |
| Susan Niermeyer, MD | Co-investigator | American Academy of Pediatrics | Dr. Niermeyer is a pediatrician/neonatologist practicing at the University of Colorado who has over 25 years experience in the design and implementation of educational programs in neonatal resuscitation to improve global neonatal survival. | Dr. Niermeyer provides technical assistance on study design, training and data collection related to the Helping Babies Breathe component of the intervention. |
| David Serukka, MD | Co-investigator | Prefa | Dr. Serukka is a public health physician with 18 years’ experience in providing reproductive health and HIV/AIDS services in both facility and community based settings in Uganda. | Dr. Serukka will supervise Ms. Namugerwa and provide technical assistance regarding study implementation. |
| Innocent Atukunda | Co-investigator | Jhpiego | Ms. Atukunda is a public health specialist with over 9 years experience in research, monitoring and evaluation of maternal and child health, HIV/AIDS, and orphans and other vulnerable children programs. | Ms. Atukunda will manage all aspects of the data collection and entry. |
| Peter Johnson, CNM | Co-investigator | Jhpiego | Dr. Johnson is a professional midwife and educational psychologist with nearly 30 years of experience in maternal health, higher education and health workforce training. | Dr. Johnson provides technical assistance related to the implementation of the training. |
| Emma Williams, MHS | Co-investigator | Jhpiego | Ms. Williams has 9 years of experience supporting research and evaluation studies in international settings. | Ms. Williams assists with design of data collection instruments, data analysis and reporting of findings. |

1. **Creation of a biospecimen repository:** Not applicable
2. **Data Coordinating Center:** Not applicable

**References**

1. Oyelese Y, Ananth CV. Postpartum hemorrhage: epidemiology, risk factors, and causes. Clin Obstet Gynecol. 2010 Mar;53(1):147–56.

2. Khan KS, Wojdyla D, Say L, Gülmezoglu AM, Van Look PFA. WHO analysis of causes of maternal death: a systematic review. Lancet. 2006 Apr 1;367(9516):1066–74.

3. Calvert C, Thomas SL, Ronsmans C, Wagner KS, Adler AJ, Filippi V. Identifying regional variation in the prevalence of postpartum haemorrhage: a systematic review and meta-analysis. Plos One. 2012;7(7):e41114.

4. Lawn JE, Lee ACC, Kinney M, Sibley L, Carlo WA, Paul VK, et al. Two million intrapartum-related stillbirths and neonatal deaths: where, why, and what can be done? Int J Gynaecol Obstet Off Organ Int Fed Gynaecol Obstet. 2009 Oct;107 Suppl 1:S5–18, S19.

5. Accountability for Maternal, Newborn and Child Survival: the 2013 Update. Geneva: World Health Organization and UNICEF; 2013.

6. Kinney MV, Kerber KJ, Black RE, Cohen B, Nkrumah F, Coovadia H, et al. Sub-Saharan Africa’s mothers, newborns, and children: where and why do they die? Plos Med. 2010 Jun;7(6):e1000294.

7. The State of the World’s Midwifery 2011. Geneva: UNFPA; 2011.

8. Helping Babies Breathe [Internet]. [cited 2013 Jun 6]. Available from: http://www.helpingbabiesbreathe.org/implementationguide.html

9. Goudar SS, Somannavar MS, Clark R, Lockyer JM, Revankar AP, Fidler HM, et al. Stillbirth and newborn mortality in India after helping babies breathe training. Pediatrics. 2013 Feb;131(2):e344–352.

10. Msemo G, Massawe A, Mmbando D, Rusibamayila N, Manji K, Kidanto HL, et al. Newborn mortality and fresh stillbirth rates in Tanzania after helping babies breathe training. Pediatrics. 2013 Feb;131(2):e353–360.

11. Carlo WA, Goudar SS, Jehan I, Chomba E, Tshefu A, Garces A, et al. Newborn-Care Training and Perinatal Mortality in Developing Countries. N Engl J Med. 2010;362(7):614–23.

12. Waiswa P, Kallander K, Peterson S, Tomson G, Pariyo GW. Using the three delays model to understand why newborn babies die in eastern Uganda. Trop Med Int Heal Tm Ih. 2010 Aug;15(8):964–72.

13. Begley CM, Gyte GM, Devane D, McGuire W, Weeks A. Active versus expectant management for women in the third stage of labour. Cochrane Database Syst Rev [Internet]. John Wiley & Sons, Ltd; 1996 [cited 2013 Jun 6]. Available from: http://onlinelibrary.wiley.com.ezproxy.welch.jhmi.edu/doi/10.1002/14651858.CD007412.pub3/abstract

14. WHO recommendations for the prevention and treatment of postpartum haemorrhage. Geneva: World Health Organization; 2012.

15. Gülmezoglu AM, Lumbiganon P, Landoulsi S, Widmer M, Abdel-Aleem H, Festin M, et al. Active management of the third stage of labour with and without controlled cord traction: a randomised, controlled, non-inferiority trial. Lancet. 2012 May 5;379(9827):1721–7.

16. Active Management of the Third Stage of Labor Data Obtained from National Survey in Uganda, April to May 2007. Washington DC: POPPHI; 2010.

17. Plotkin, Tibaijuka, Makene, Currie, Lacoste. Quality of Care for Prevention and Treatment of Common Maternal and Newborn Complications: A study of 12 regions in Tanzania [Internet]. USAID and MCHIP; 2011. Available from: http://www.mchip.net

18. Kagame, Ricca, Rawlins, Rosen, Lynam, Kidula. Quality of Care for Prevention and Management of Common Maternal and Newborn Complications: Findings from a National Health. Facility Survey in Kenya. Are Services Provided According to International Standards? [Internet]. USAID and MCHIP; 2011. Available from: http://www.mchip.net

19. Althabe F, Buekens P, Bergel E, Belizán JM, Campbell MK, Moss N, et al. A behavioral intervention to improve obstetrical care. N Engl J Med. 2008 May 1;358(18):1929–40.

20. Bluestone, Julia, Johnson, Peter, Fullerton, Judith, Carr, Catherine, Alderman, Jessica, BonTempo, James. Effective In-Service Training Techniques, Frequency, Setting and Media: Evidence from Integrative Review of the Literature. Baltimore: Jhpiego; 2012.

21. Zurovac D, Sudoi RK, Akhwale WS, Ndiritu M, Hamer DH, Rowe AK, et al. The effect of mobile phone text-message reminders on Kenyan health workers’ adherence to malaria treatment guidelines: a cluster randomised trial. Lancet. 2011 Aug 27;378(9793):795–803.

22. Piaggio G, Elbourne DR, Pocock SJ, Evans SJW, Altman DG, CONSORT Group. Reporting of noninferiority and equivalence randomized trials: extension of the CONSORT 2010 statement. Jama J Am Med Assoc. 2012 Dec 26;308(24):2594–604.

1. Note: These rates or proportions may increase between baseline and midline due to better reporting, and we expect these to decrease due to the intervention between midline and endline. [↑](#footnote-ref-2)
